# Proteomic profile of extracellular vesicles from plasma and CSF of multiple sclerosis patients reveals disease activity-associated EAAT2

**DOI:** 10.1186/s12974-024-03148-x

**Published:** 2024-09-02

**Authors:** Antonella D’Ambrosio, Silvia Zamboni, Serena Camerini, Marialuisa Casella, Massimo Sanchez, Donatella Pietraforte, Nicola Vanacore, Marco Diociauti, Marta Altieri, Vittorio Di Piero, Ada Francia, Simona Pontecorvo, Marco Puthenparampil, Paolo Gallo, Paola Margutti

**Affiliations:** 1 Department of Neuroscience, Istituto Superiore di Sanità, Vle Regina Elena 299, 00161 Rome, Italy; 2 Core Facilities, Istituto Superiore di Sanità, 00161 Rome, Italy; 3 Center of Disease Prevention and Health Promotion, Istituto Superiore di Sanità, 00161 Rome, Italy; 4 Department of Human Neurosciences, University “La Sapienza”, 00185 Rome, Italy; 5 Department of Neurosciences, University of Padua, 35128 Padua, Italy

**Keywords:** Multiple sclerosis, Extracellular vesicles, Comparative proteomics, Synaptic transmission pathway, Disease activity

## Abstract

**Background and objectives:**

There is an urgent need to discover blood-based biomarkers of multiple sclerosis (MS) to better define the underlying biology of relapses and monitor disease progression. The main goal of this study is to search for candidate biomarkers of MS relapses associated with circulating extracellular vesicles (EVs), an emerging tool for biomarker discovery.

**Methods:**

EVs, purified from unpaired plasma and CSF samples of RRMS patients by size-exclusion chromatography (SEC), underwent proteomic analysis to discover novel biomarkers associated with MS relapses. The candidate biomarkers of disease activity were detected by comparison approach between plasma- and CSF-EV proteomes associated with relapses. Among them, a selected potential biomarker was evaluated in a cohort of MS patients, using a novel and highly reproducible flow cytometry-based approach in order to detect low abundant EV subsets in a complex body fluid such as plasma.

**Results:**

The proteomic profiles of both SEC-purified plasma EVs (from 6 patients in relapse and 5 patients in remission) and SEC-purified CSF EVs (from 4 patients in relapse and 3 patients in remission) revealed a set of proteins associated with MS relapses significant enriched in the synaptic transmission pathway. Among common proteins, excitatory amino-acid transporter 2, EAAT2, responsible for the majority of the glutamate uptake in CNS, was worthy of further investigation. By screening plasma samples from 110 MS patients, we found a significant association of plasma EV-carried EAAT2 protein (EV-EAAT2) with MS relapses, regardless of disease-modifying therapies. This finding was confirmed by investigating the presence of EV-EAAT2 in plasma samples collected longitudinally from 10 RRMS patients, during relapse and remission. Moreover, plasma EV-EAAT2 levels correlated positively with Expanded Disability Status Scale (EDSS) score in remitting MS patients but showed a negative correlation with age in patients with secondary progressive (SPMS).

**Conclusion:**

Our results emphaticize the usefulness of plasma EVs as a source of accessible biomarkers to remotely analyse the CNS status. Plasma EV-EAAT2 showed to be a promising biomarker for MS relapses. Further studies are required to assess the clinical relevance of this biomarker also for disability progression independent of relapse activity and transition from RRMS towards SPMS.

**Supplementary Information:**

The online version contains supplementary material available at 10.1186/s12974-024-03148-x.

## Introduction

Multiple sclerosis (MS) is a chronic demyelinating and neurodegenerative disease of the CNS affecting young adults, that typically manifests with episodes of transient exacerbations of neurological disability (relapses), followed by partial or total recovery [1]. MS relapses, a key feature of relapsing–remitting multiple sclerosis (RRMS), the most prevalent MS phenotype, are defined as occurrence of new symptoms or worsening of old symptoms, not always accompanied by the detection of contrast-enhancing lesions, by MRI. This may be due at least in part to the limited sensitivity of conventional MRI to detect small lesions, particularly in the spinal cord, cortical grey matter and optic nerve [2–4]. In the context of clinical sign and symptom worsening, in the absence of the gadolinium-enhancing lesions, and aiming to improve treatment decisions, it is important to distinguish MS relapses from pseudo-relapses, which may be triggered by infection or comorbidities [5]. On the other hand, MRI may reveal active lesions without symptoms, indicating a subclinical relapse [6]. Recent data have shown the impact of effective prevention of relapses on long-term disability [7]. However, there is also evidence of disability accumulation unrelated to relapses in RRMS; this condition, referred to as progression independent of relapse activity (PIRA), is associated with a predominant underlying neurodegenerative component [8].

Given the heterogeneity of MS course and the increasing number of disease-modifying therapies (DMTs) for RRMS, with different safety profiles and efficacy in reducing CNS inflammation and relapse rates, the discovery of peripheral biomarkers that facilitate disease activity assessment and personalized treatment, would greatly improve patient care.

Currently, serum  neurofilament-light chain (sNfl) and glial fibrillary acidic protein (GFAP) have shown promise as biomarkers of acute disease activity and progression [9, 10]. However, there are several limitations to the potential use of these molecules as peripheral biomarkers mainly due to confounding factors, such as age [11]. Therefore, there is an emerging interest in searching for novel MS biomarkers in order to develop a panel of molecules that might be used in the clinical practice.

Growing evidence in neurological diseases indicates extracellular vesicles (EVs), an heterogeneous family of extracellular structures bounded by a phospholipid bilayer, released by all CNS cell types in cerebrospinal fluid (CSF), as vehicles of intercellular communication involved in many physiological and pathological processes [12]. The ability of CNS-derived EVs to cross the blood–brain barrier (BBB) and entering the peripheral blood, makes them an easily accessible biomarker source of neurological disorders, including MS [13]. Moreover, EVs, sharing the same antigenic repertoire as their parental cells, may dynamically reflect the pathologic mechanisms underlying CNS damage.

Therefore, EVs, with their molecular constituents more stable than soluble molecules in body fluids, are becoming object of multi-omics investigation not only to reveal novel biomarkers of the disease but also to improve the knowledge of the molecular mechanisms underpinning MS pathogenesis. In this study, we investigated the proteome composition of plasma and CSF EVs obtained from RRMS patients aiming at identifying potential peripheral biomarkers associated with disease activity. We have selected one plasma candidate biomarker associated with relapses and established a novel flow cytometry-based assay for its detection in a larger MS cohort, including RRMS and SPMS patients.

## Materials and methods

### Study population

In this multi-center longitudinal study, 110 patients with a diagnosis of MS based on the 2018 revised McDonald criteria [14], were enrolled at the Department of Neuroscience, ‘La Sapienza’ University of Rome, and the Department of Neuroscience, University of Padua, Italy, between 2017 and 2019. The study included 83 RRMS patients (41 with no clinically or radiologically evident relapse for at least 12 months and 42 in acute relapse) and 27 SPMS patients (Table 1). Inclusion criteria for patient enrollment were: age from 18 to 65 years; no comorbidities or infectious diseases and no steroid therapy in the month before blood sampling; no women in pregnancy, lactation, or planning a pregnancy. Patient characterization included a clinical evaluation with EDSS score and MRI assessment. The control group included 23 sex- and age-matched healthy controls. This study was approved by the ethics committees of the ‘La Sapienza’ University of Rome (725/16) and Istituto Superiore di Sanità (174/16). Signed informed consent was obtained from all the enrolled study subjects.

**Table 1:** Demographic and clinical characteristics of MS patients and controls

		RRMS Relapse	RRMS Remission	SPMS	HC	Total
*Demographic characteristics*						
No. of patients		42	41	27	23	133
Gender	Female/male	26/16	30/11	15/12	12/11	83/50
Age	Mean (SD)	40,9 ± 10,2	44,3 ± 11,2	53,6 ± 7,6	44,9 ± 14,7	45,2 ± 11.8
	Range (years)	21–62	24–62	35–65	22–65	21–65
*Clinical*						
Disease Duration	Mean (SD)	3.1 ± 3.5	6.9 ± 4.4	15 ± 7.3	–	7.4 ± 6.8
	Range (years)	0–12	1–16	5–25	–	0–25
EDSS score	Mean (SD)	1,2 ± 0,75	1.4 ± 1.02	4,6 ± 1,8	–	2.1 ± 1.8
	Range	0–3	0–4	- 8	–	0–8
Age at disease onset	Mean (SD)	37,8 ± 10,1	37,4 ± 9,6	39 ± 10,3	–	–
(DMTs) yes/no		15/27	24/17	0/27	–	

EDSS: Expanded Disability Status Scale

DMTs: Disease Modifying Therapies

### Blood and CSF samples

Blood samples were collected in acid citrate/dextrose tubes (Becton Dickinson, vacutainer ACD solution B) and processed within 60 min from collection to obtain Platelet-Poor Plasma (PPP). In particular, platelet removal was performed by using two subsequent centrifugations at 2500*g* for 15 min., as recommended by the International Society on Thrombosis and Haemostasis (ISTH) [15].

CSF specimens were collected by non-traumatic lumbar puncture as previously reported, and centrifuged at 2000*g* for 15 min. to remove residual cells and other insoluble material [16]. All PPP and CSF samples were aliquoted and kept frozen at − 80 °C until use. Among 83 RRMS patients enrolled in this study, plasma samples from 10 patients were collected at relapse and remission, over a one year period. For EV proteomic analysis, plasma samples from 11 untreated RRMS patients (6 in relapse and 5 in remission) and 5 healthy controls and CSF samples from 7 untreated RRMS patients (4 in relapse and 3 in remission) were used. For flow cytometry analysis, plasma samples from RRMS patients with (n = 39) or without (n = 44) first-line DMTs for at least 3 months from blood sampling, and 27 untreated SPMS patients, were used. DMTs included interferons, teriflunomide, glatiramer acetate or dimethyl fumarate.

### Size-exclusion chromatography (SEC)

#### SEC plasma EV purification

For purification of plasma EVs by size, individual PPP (6 ml) samples were loaded on to a Sephacryl S-500 gel filtration column (GE Healthcare). The SEC-fractions were centrifugated at 20.000*g* for 2 h and the pellets were washed and analysed for determination of protein concentration using Bradford protein assay. After that, the supernatants of the SEC-fractions were further centrifuged at 100.000*g* for 2 h and the pellets obtained were washed and evaluated for determination of protein concentration. Moreover, the pellets with the major content of proteins obtained after the two centrifugations, were analysed by Nanosight for determination of particle-size distribution and concentration before to be pooled together (SEC-purified plasma EVs) for TEM and proteomic analysis. For a detailed protocol about SEC plasma EV purification, see Supplementary methods.

#### SEC CSF EV purification

For purification of CSF EVs by size, individual CSF samples (4 ml) were loaded on to a Sephacryl S-500 gel filtration column (GE Healthcare). The SEC-fractions were centrifuged at 100.000*g* for 2 h and the pellets were washed and pooled together for TEM and proteomic analysis. For a detailed protocol about SEC CSF EV purification, see Supplementary methods.

### Nanoparticle tracking analysis

The number and size of SEC-purified plasma EVs were assessed by nanoparticle tracking analysis (NTA) (NanoSight Model NS300, Malvern Instruments, NanoSight Ltd., Salisbury, United Kingdom). The parameters for NTA capture setting were as follows: camera type (sCMOS), Laser type Blue488, capture level 15, threshold 5, slider gain (366), and capture duration (60 s). Five videos of typically 60 s duration were taken. Data were analyzed by NTA 3.0 software (Malvern Instruments), which was optimized to first identify and then track each particle on a frame-by-frame basis.

### Transmission electron microscopy (TEM)

SEC-purified plasma and CSF EVs were deposited and dried onto thin substrates of amorphous carbon and negatively stained with 2% (w/v) phosphotungstic acid. Samples were observed using a Zeiss EM902 transmission electron microscope, operating at 80 kV and equipped with an “in column” electron energy filter. Images were acquired with a digital charge-coupled device camera, model PROSCAN HSC2 (1 K Å ~ 1 K pixels), thermostated by a Peltier unit. Image analysis was performed using the digital analyzer SIS 3.0 and the overall resolution can be estimated in the order of 2 nm.

### Proteomic analysis

SEC-purified EVs were loaded on 1D-gel NuPAGE 4–12% and trypsin digested in 10 contiguous slices cut in each gel lane [17]. The resulting peptide mixtures were separated by an Ultimate 3000 HPLC (DIONEX, USA) connected with a linear ion trap mass spectrometer (LTQ-XL, ThermoElectron, USA): they were desalted on a trap column (Acclaim PepMap 100 C18, LC Packings, DIONEX) and separated on a 10 cm long column (Silica Tips FS 360-75-8, New Objective, USA) slurry-packed in-house with 5 µm, 200 Å pore size C18 resin (Michrom BioResources, USA). A 50 min gradient from 4 to 80% buffer B (95% acetonitrile and 0.1% formic acid) and buffer A(5% acetonitrile and 0.1% formic acid) was used at 300 nL/min flow rate. MS spectra were acquired from 400 to 2000 m/z in a Top 5 data-dependent mode, with 45 s long dynamic exclusion and applying 35% CID for fragmentation. Tandem mass spectra were matched against the Homo sapiens protein database (http://www.uniprot.org/downloads) and through Bioworks software (version 3.3, Thermo Electron). Fully tryptic cleavage constraints (one miss-cleavage allowed), static cysteine carbamidomethylation, and variable methionine oxidation were considered as match parameters and 1.5 and 1 Da were used as mass tolerance for precursor and fragment ions, respectively. For peptide identification cross correlation scores of 1.8, 2.5 and 3 for 1, 2 and 3 peptide charge state, respectively, and peptide probability cut-off of P < 0.001 were used. Proteins were identified with at least two peptides.

### Western blot analysis

To assess the quality of purified plasma EV samples, SEC-purified plasma EVs after 20.000*g* centrifugation (SEC-EVs 20K) and SEC-purified plasma EVs after 100.000*g* centrifugation (SEC-EVs 100K), were used for western blot analysis using antibodies specific for exosome and lipoprotein markers (Supplementary methods).

To assess the presence of EAAT2 protein, SEC-purified plasma EVs, SEC-EVs 20K and SEC-EVs 100K as well as EVs isolated by centrifugation (100.000*g* for 20 min) from the culture supernatant of U251 multiform glioblastoma and chronic myelogenous leukemia K562 cell lines were used. For each sample 20 µg of proteins, determined using the Bradford protein assay (Bio-Rad, USA), was loaded on polyacrylamide gels. Proteins were separated on 10% pre-casted acrylamide gels (Invitrogen, Carlsbad, CA) and transferred to PVDF membranes. The membranes were blocked (5% milk and 0.05% Tween-20) for 2 h and incubated overnight with EAAT2-specific rabbit polyclonal antibody (1 µg) (Bioss, USA), as primary antibody. After washing in PBS, the secondary HRP-conjugated anti-rabbit IgG (Sigma-Aldrich) was added. Chemiluminescent detection of proteins was performed using ECL Plus reagent (Amersham).

### Flow cytometry gating strategy

EVs in PPP samples were analyzed using Gallios flow cytometer (Beckman Coulter, USA) after an accurate setting of the physical and fluorescence parameters. In particular, for the correct setting of the gate (based on the size of EVs) and the fluorescence parameters, fluorescent beads of variable size were used (Flow Cytometry Sub-micron Particle Size Reference Kit- Thermo Fischer scientific). The flow cytometer was adjusted to cover the EV size range between 0.5 and 1 µm. Moreover, FCS threshold value was determined to reduce the background noise of the instrument preserving the detection of the EV population of 0.5 µm in size. A routine verification of optical alignment of lasers and fluidic stability of flow cytometer were performed daily with Flow-Check Pro Fluorosphere (Beckman Coulter), according to the manufacturer's instructions. The correct setting of 0.5–1 µm range size for EVs gate was periodically checked. Data were analyzed using Kaluza software 1.2 (Beckman Coulter).

### Identification of EVs by MTG labelling

SEC-purified plasma EVs or EVs in PPP samples were labeled with MITO Tracker Green FM (MTG) (Molecular Probes-Invitrogen) to identify EV populations using a newly developed flow cytometry assay described in Supplementary methods. Since EVs and lipoproteins have overlapping particle size distribution in plasma, MTG propriety to discern between EVs and large lipoproteins in 0.5–1 µm gate (in flow cytometry) was assessed by using purified chylomicrons and very low-density lipoproteins (Supplementary methods).

### EAAT2 labelling of EVs

Flow Count Fluorosphere (Beckman Coulter) with a known number of fluorescent beads were utilized for EV quantification, according to manufacturer’s instructions. A volume of PPP containing 1 × 10^6^ EVs was diluted in PBS and the analysis of the EAAT2 protein on the EV surface in plasma samples of MS patients and healthy controls was performed using 1 µg of PE-conjugated EAAT2-specific rabbit polyclonal antibody (Bioss, USA) for 45 min at RT. Then, MTG (100 nM) was added for 15 min at RT before FACS acquisition. To avoid immune complex formation and the unspecific background due to antibody aggregation, each antibody and reagent was centrifuged before use (20.000*g* for 20 min) [18]. The amount of antibody used for EV staining was titrated in order to determine the optimal concentration and have a low signal-to-noise ratio. Unstained EVs and/or uncorrelated matching antibody isotype (Bioss Rabbit IgG isotype control, PE conjugated, USA), were used to determine the background fluorescence. The flow cytometry results were expressed as percentage of EVs carrying EAAT2 protein (EV-EAAT2) in EV MTG-positive gate. EAAT2 measurement was made in triplicate for each sample and the mean values with a standard deviation (SD) less than 10% were used for data analysis.

### EAAT2 detection on EVs derived from U251 and K562 cell lines

U251 cell line, provided by Calogero [19], and K562 cell line culture conditions and EV isolation from supernatants are reported in Supplementary methods.

### Statistical and data analysis

Kolmogorov–Smirnov’s and Shapiro–Wilk’s tests to evaluate the normal distribution of the data and non-parametric tests were used. In particular, to compare data obtained in different patient subgroups, Kruskal–Wallis test was used. The correlation analyses were performed by Spearman's Rho. To compare two groups Mann–Whitney test was used to determine statistical significance. Wilcoxon signed-ranks test was used to assess a difference in the mean (or median) of paired observations. The p value ≤ 0.05 was considered statistically significant. The SPSS Version 28.0 and Graphpad Prism 5 software were used for statistical analyses. We annotated the identified proteins using the UniProt database (http://www.uniprot.org/). To identify the GO cellular components enriched by a set of proteins or genes, we used the Database for Annotation, Visualization and Integrated Discovery (DAVID), updated on September 22, 2023 (https://david.ncifcrf.gov/home.jsp). In addition, we performed the Functional enrichment 3.1.3 (FunRich 3.1.3) and REACTOME pathway analysis (https://reactome.org/) (Pathan et al. 2015). For KEGG pathway analysis, ShinyGO v0.741 (http://bioinformatics.sdstate.edu/go74/) was used.

## Results

**Fig. 1:**
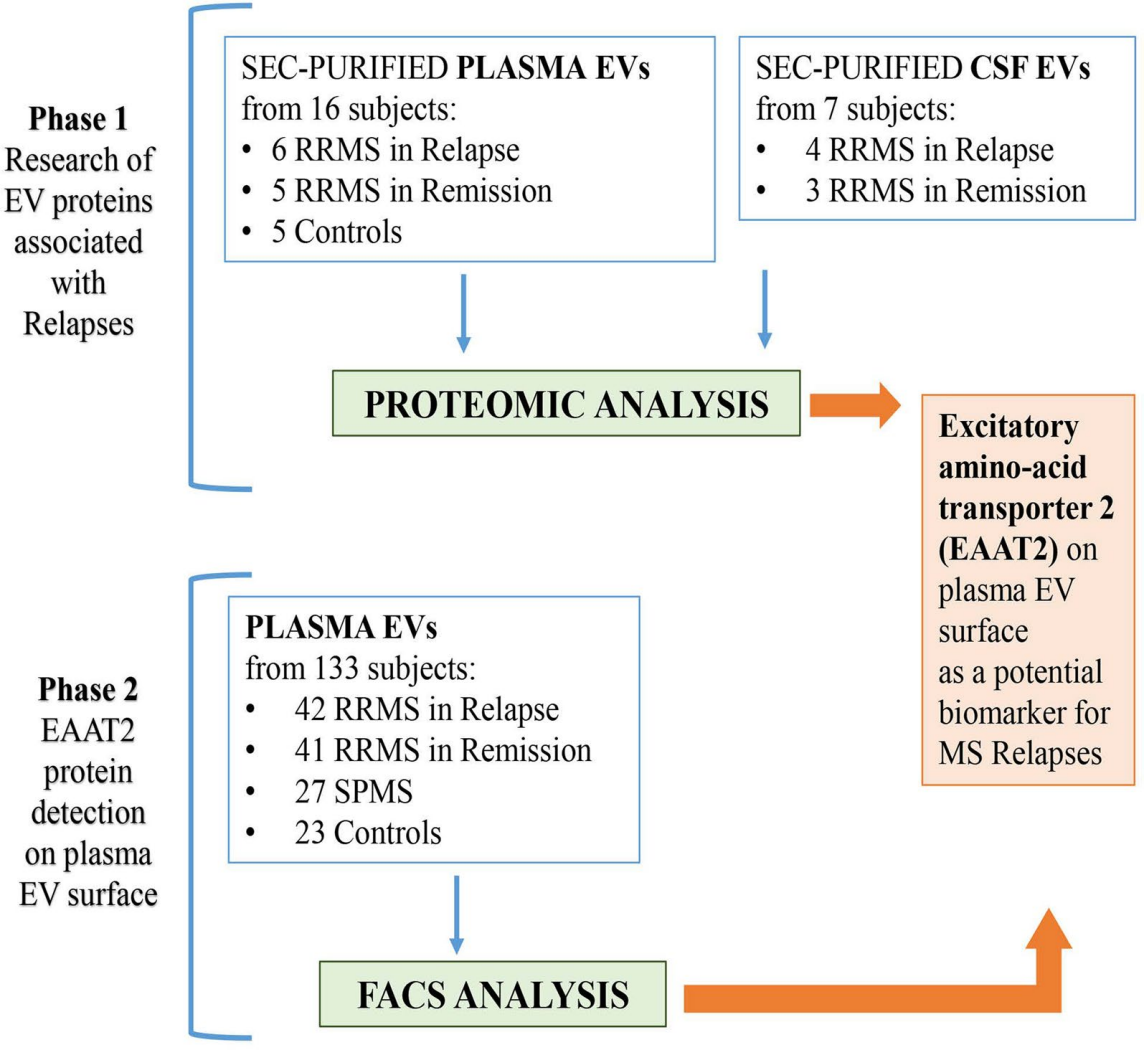
Schematic representation of study design

Aiming at identifying peripheral biomarkers associated with MS relapse, as a first step we investigated the proteomic profile of EVs purified from unpaired plasma and CSF samples of RRMS patients. The complete study design is depicted in Fig. 1.

### Proteomic characterization of plasma EVs

EVs were purified from plasma samples of 11 RRMS patients (6 patients in relapse and 5 patients in remission) and 5 healthy controls by SEC. To evaluate the goodness of plasma EV purification strategy adopted in this study, purified plasma EV samples were characterized for exosome and lipoprotein markers, as reported in Supplementary results.

**Fig. 2:**
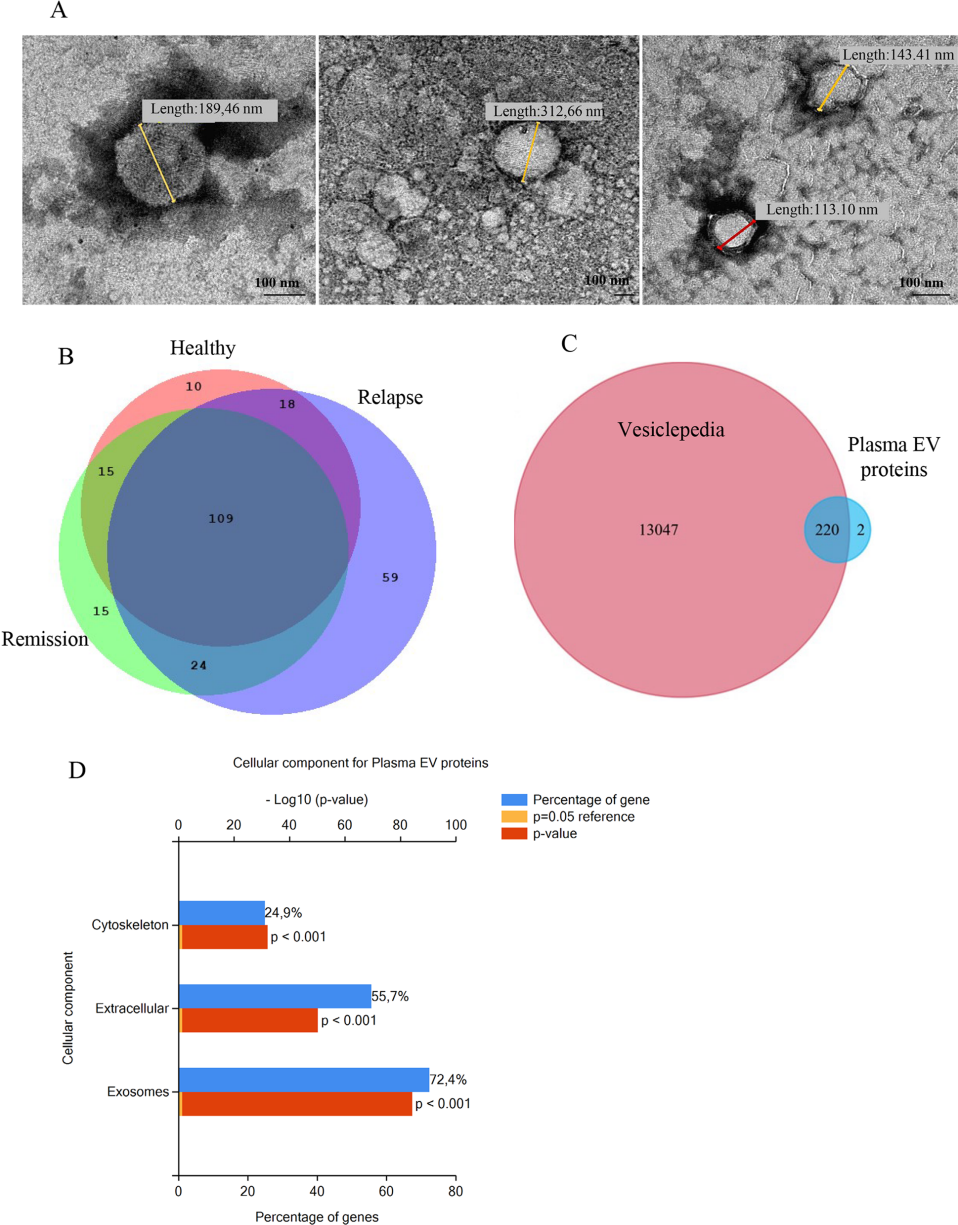
Characterization of SEC-purified plasma EVs. **A** Representative images of SEC-purified plasma EVs obtained with TEM. **B** Venn diagram showing the number of common and unique proteins in relapsing and remitting MS patients and healthy controls. **C** Venn diagram showing the total identified proteins compared with Vesiclepedia database. **D** Functional gene enrichment analysis of all identified proteins from the FunRich software for cellular component (*p* ≤ 0.001)

SEC-purified plasma EVs visualized by TEM, appeared as lipid bilayer enclosed particles that ranged in size from 50 to 700 nm, confirming the validity of the EV isolation protocol (Fig. 2A). Proteomic analysis of SEC-purified plasma EVs was carried out through pre-fractionation of samples by one dimensional SDS-PAGE followed by liquid chromatography-tandem mass spectrometry (LCMS/MS). A repertoire of 250 proteins were identified (Supplementary Table 1) and distributed as indicated in the Venn diagram (Fig. 2B). The protein list derived from each group is reported in Supplementary Tables 2, 3 and 4. Furthermore, the matrix charts, showing pairwise comparison of shared EV proteins between subjects of the same group, are reported in Supplementary Fig. 2A, 2B and 2C.

FunRich 3.1.3 analysis relative to cellular structures and comparison with Vesiclepedia database showed that the total proteins identified in plasma EVs were significantly enriched in extracellular vesicles (*p* ≤ 0.001) (Fig. 2D and C).

**Fig. 3:**
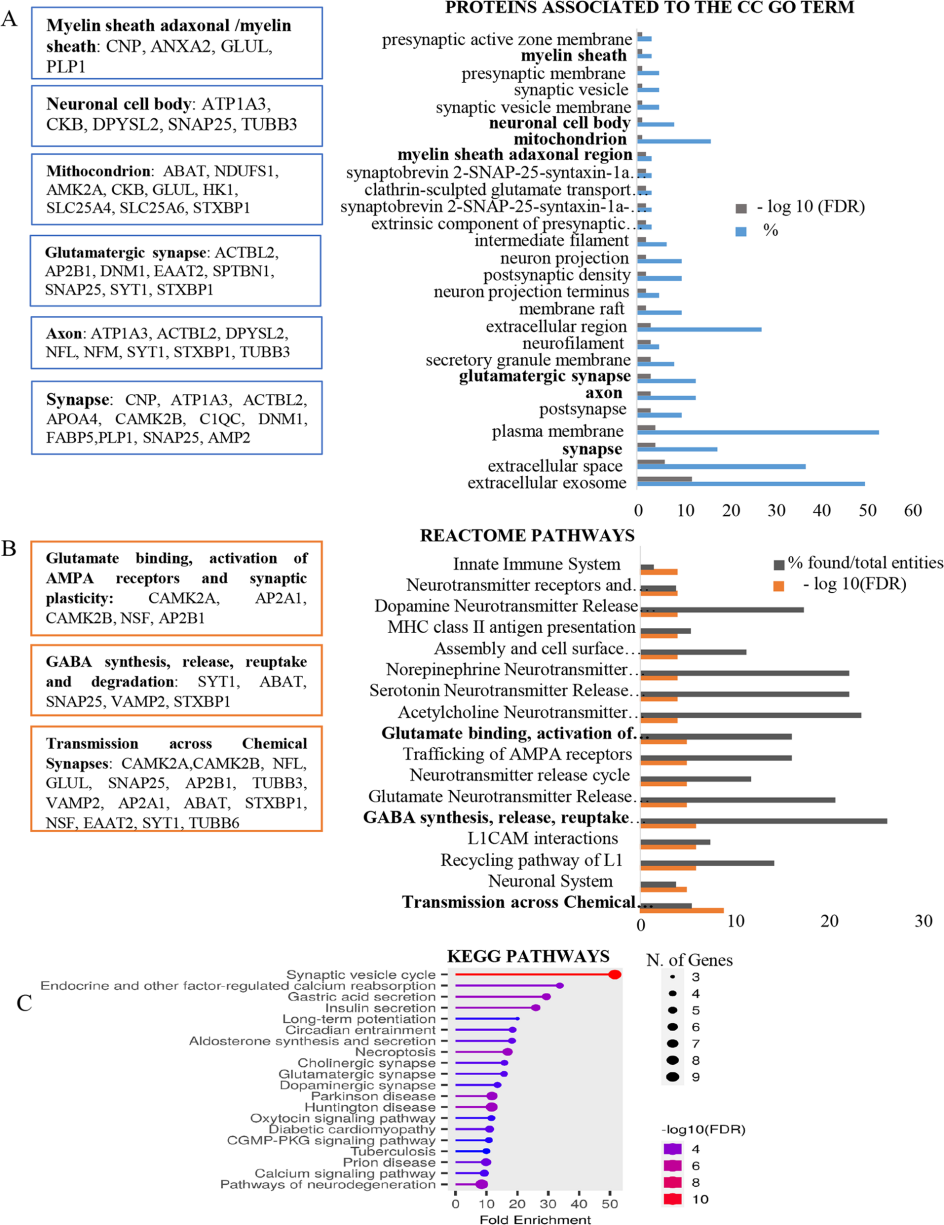
Proteome profiling of plasma-derived EVs associated with MS relapses. **A** Cellular Component (CC) GO term enrichment analysis for unique 59 plasma EV proteins associated with the relapsing phase. In the boxes, proteins linked to various components obtained by DAVID bioinformatics tool, are listed. **B**, **C** Pathways identified by using Reactome and KEGG database, respectively. In the boxes of the (**B**) panel, the list of proteins associated with the most significant Reactome pathways, are listed

As shown in Venn diagram (Fig. 2B), comparison of the EV proteomes among the three groups analysed (relapsing patients, remitting patients and healthy controls) revealed 59 unique proteins associated with the relapsing phase of MS (Table 2). By means of DAVID analysis, these 59 proteins, classified into the Cellular Component Gene Ontology (CC GO) term, were significantly enriched in proteins present in the synapsis, axon, mitochondrion, neuronal cell body and myelin sheath (Fig. 3A). KEGG pathway analysis revealed that most of these proteins are associated with synaptic vesicle cycle, in line with the results of Reactome Pathway analysis, showing the involvement of this protein set in the synaptic transmission pathway (Fig. 3B and C).

**Table 2:** Lists of unique proteins of plasma- and CSF-derived EVs associated with MS relapse

	Plasma EV proteins associated with relapses		
	Acc. number	Gene names	Protein names
1	P09543	CNP	2′,3′-cyclic-nucleotide 3′-phosphodiesterase
2	P80404	ABAT,	4-aminobutyrate aminotransferase, mitochondrial
3	P12235	SLC25A4	ADP/ATP translocase 1
4	P12236	SLC25A6	ADP/ATP translocase 3
5	P43652	AFM	Afamin
6	A8K2U0	A2ML1	Α-2-macroglobulin-like protein 1
7	Q16352	INA	Α-internexin
8	P15144	ANPEP,	Aminopeptidase N
9	P07355	ANXA2	Annexin A2
10	O95782	AP2A1,	AP-2 complex subunit α-1
11	P63010	AP2B1,	AP-2 complex subunit β
12	P06727	APOA4	Apolipoprotein A-IV
13	P02656	APOC3	Apolipoprotein C-III
14	Q9UKV3	ACIN1	Apoptotic chromatin condensation inducer in the nucleus
15	Q562R1	ACTBL2	Β-actin-like protein 2
16	Q9UQM7	CAMK2A	Calcium/calmodulin-dependent protein kinase type II subunit α
17	Q13554	CAMK2B	Calcium/calmodulin-dependent protein kinase type II subunit β
18	P07858	CTSB	Cathepsin B
19	P07339	CTSD	Cathepsin D
20	P02747	C1QC	Complement C1q subcomponent subunit C
21	Q03591	CFHR1	Complement factor H-related protein 1
22	P02741	CRP	C-reactive protein
23	P12277	CKB	Creatine kinase B-type
24	Q16555	DPYSL2	Dihydropyrimidinase-related protein 2
25	O95147	DUSP14	Dual specificity protein phosphatase 14
26	Q05193	DNM1	Dynamin-1
27	P43004	EAAT2	Excitatory amino acid transporter 2
28	P15311	EZR	Ezrin
29	Q01469	FABP5	Fatty acid-binding protein 5
30	P14136	GFAP	Glial fibrillary acidic protein
31	P15104	GLUL, GLNS	Glutamine synthetase
32	P09471	GNAO1	Guanine nucleotide-binding protein G(o) subunit α
33	P19367	HK1	Hexokinase-1
34	P04908	H2AC4	Histone H2A type 1-B/E
35	P33778	H2BC3	Histone H2B type 1-B
36	P01762	IGHV3-11	Immunoglobulin heavy variable 3–11
37	P01614	IGKV2D-40	Immunoglobulin kappa variable 2D-40
38	Q14643	ITPR1	Inositol 1,4,5-trisphosphate receptor type 1
39	P29622	SERPINA4,	Kallistatin
40	P60201	PLP1	Myelin proteolipid protein
41	P28331	NDUFS1	NADH-ubiquinone oxidoreductase 75 kDa subunit, mitochondrial
42	P07196	NFL	Neurofilament light polypeptide
43	P07197	NFM	Neurofilament medium polypeptide
44	P15309	ACP3	Prostatic acid phosphatase
45	P06702	S100A9	Protein S100-A9
46	P22735	TGM1	Protein-glutamine gamma-glutamyltransferase K
47	P13637	ATP1A3	Sodium/potassium-transporting ATPase subunit α-3
48	Q13813	SPTAN1	Spectrin α chain, non-erythrocytic 1
49	Q01082	SPTBN1	Spectrin β chain, non-erythrocytic 1
50	P60880	SNAP25	Synaptosomal-associated protein 25
51	P21579	SYT1	Synaptotagmin-1
52	P61764	STXBP1	Syntaxin-binding protein 1
53	Q5TAX3	TUT4	Terminal uridylyltransferase 4
54	P07437	TUBB	Tubulin β chain
55	Q13509	TUBB3	Tubulin β-3 chain
56	Q9BUF5	TUBB6	Tubulin β-6 chain
57	P63027	VAMP2	Vesicle-associated membrane protein 2
58	P46459	NSF	Vesicle-fusing ATPase
59	Q93050	ATP6V0A1	V-type proton ATPase 116 kDa subunit a 1

CSF EV proteins associated with relapses			
	Acc. number	Gene names	Protein names
1	P62258	YWHAE	14–3-3 protein epsilon
2	P68133	ACTA1	Actin, α skeletal muscle
3	P12235	SLC25A4	ADP/ATP translocase 1
4	P05141	SLC25A5,	ADP/ATP translocase 2
5	P61204	ARF3	ADP-ribosylation factor 3
6	P02763	ORM1	Α-1-acid glycoprotein 1
7	P08697	SERPINF2	Α-2-antiplasmin
8	P02765	AHSG	Α-2-HS-glycoprotein
9	P35609	ACTN2	Α-actinin-2
10	O94973	AP2A2	AP-2 complex subunit α-2
11	P63010	AP2B1	AP-2 complex subunit β
12	P25705	ATP5F1A	ATP synthase subunit α, mitochondrial
13	P02730	SLC4A1	Band 3 anion transport protein
14	P01031	C5	Complement C5
15	P05156	CFI	Complement factor I
16	P12277	CKB	Creatine kinase B-type
17	O75746	SLC25A12	Electrogenic aspartate/glutamate antiporter, mitochondrial
18	P43004	EAAT2	Excitatory amino acid transporter 2
19	Q15485	FCN2	Ficolin-2
20	P14136	GFAP	Glial fibrillary acidic protein
21	P09471	GNAO1	Guanine nucleotide-binding protein G(o) subunit α
22	P08238	HSP90AB1	Heat shock protein HSP 90-β
23	P68871	HBB	Hemoglobin subunit β
24	P05546	SERPIND1	Heparin cofactor 2
25	P19367	HK1	Hexokinase-1
26	Q14764	MVP	Major vault protein
27	Q02978	SLC25A11	Mitochondrial 2-oxoglutarate/malate carrier protein
28	P02686	MBP	Myelin basic protein
29	P12882	MYH1	Myosin-1
30	Q9UKX2	MYH2	Myosin-2
31	P11055	MYH3	Myosin-3
32	P12883	MYH7	Myosin-7
33	P13535	MYH8	Myosin-8
34	Q92823	NRCAM	Neuronal cell adhesion molecule
35	P08567	PLEK	Pleckstrin
36	P12273	PIP	Prolactin-inducible protein
37	P31151	S100A7	Protein S100-A7
38	P14618	PKM	Pyruvate kinase PKM
39	P30153	PPP2R1A	Serine/threonine-protein phosphatase 2A 65 kDa regulatory subunit A α isoform
40	P05023	ATP1A1	Sodium/potassium-transporting ATPase subunit α-1
41	P50993	ATP1A2	Sodium/potassium-transporting ATPase subunit α-2
42	P13637	ATP1A3	Sodium/potassium-transporting ATPase subunit α-3
43	P38646	HSPA9	Stress-70 protein, mitochondrial
44	P61764	STXBP1	Syntaxin-binding protein 1
45	P68363	TUBA1B	Tubulin α-1B chain
46	Q13509	TUBB3	Tubulin β-3 chain
47	P68371	TUBB4B	Tubulin β-4B chain
48	P02774	GC	Vitamin D-binding protein

### Proteomic characterization of CSF EVs

**Fig. 4:**
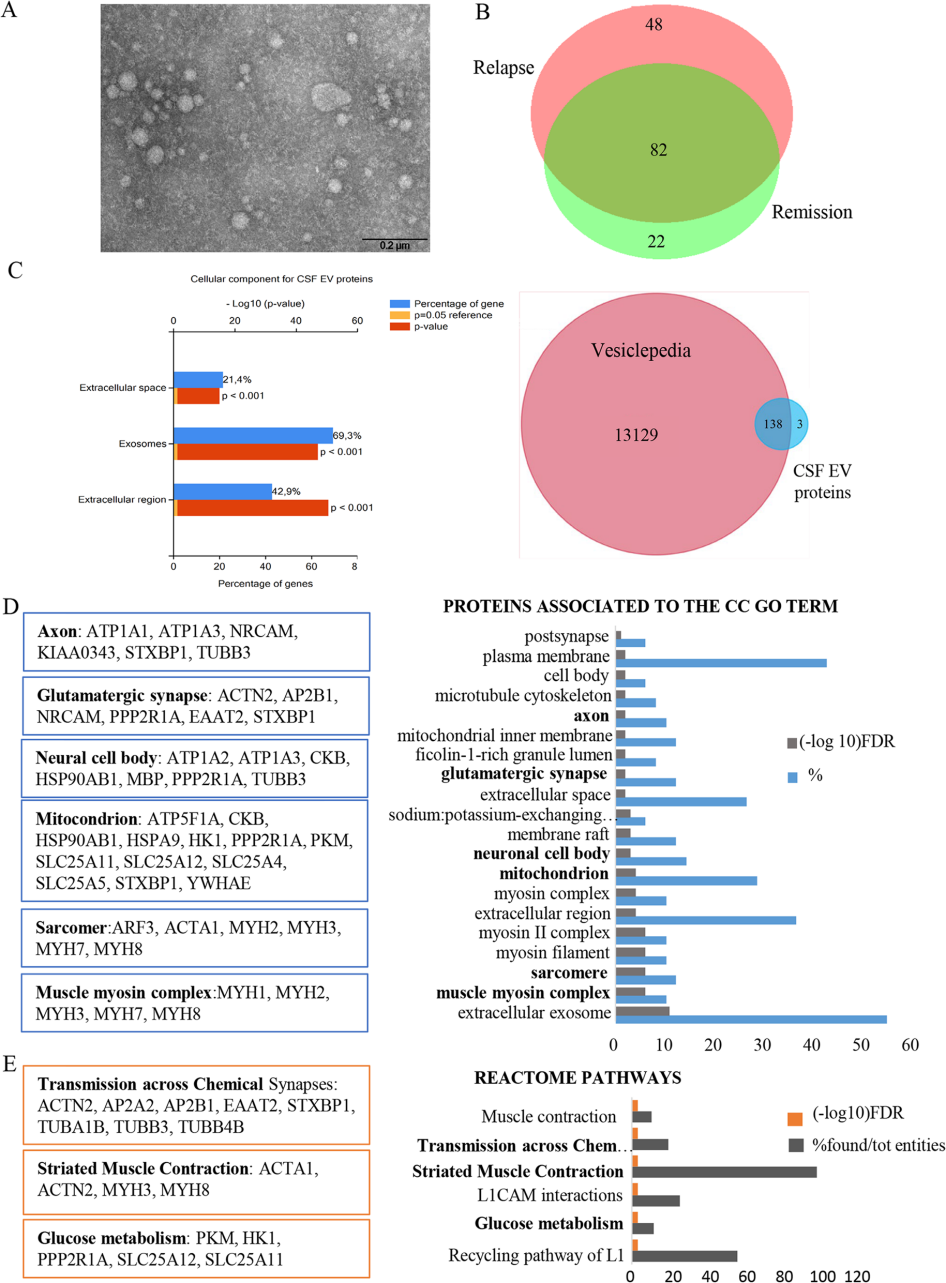
Proteome profiling of CSF-derived EVs associated with MS relapses. **A** Representative image of SEC-purified CSF EVs obtained with TEM. **B** Venn diagram showing common and unique proteins of CSF-derived EVs from relapsing and remitting MS patients. **C** FunRich functional analysis results of 152 total proteins related to cellular components; Venn diagram showing the comparison between total proteins identified in SEC-purified CSF EVs and Vesiclepedia database. **D** Cellular Component (CC) GO term enrichment analysis for unique 48 CSF EV proteins associated with relapse. In the boxes, proteins linked to some significant structure components obtained by DAVID bioinformatics tool, are listed. **E** Pathways identified using Reactome. In the boxes, proteins associated with some of the more significant Reactome pathways, are listed

**Fig. 5:**
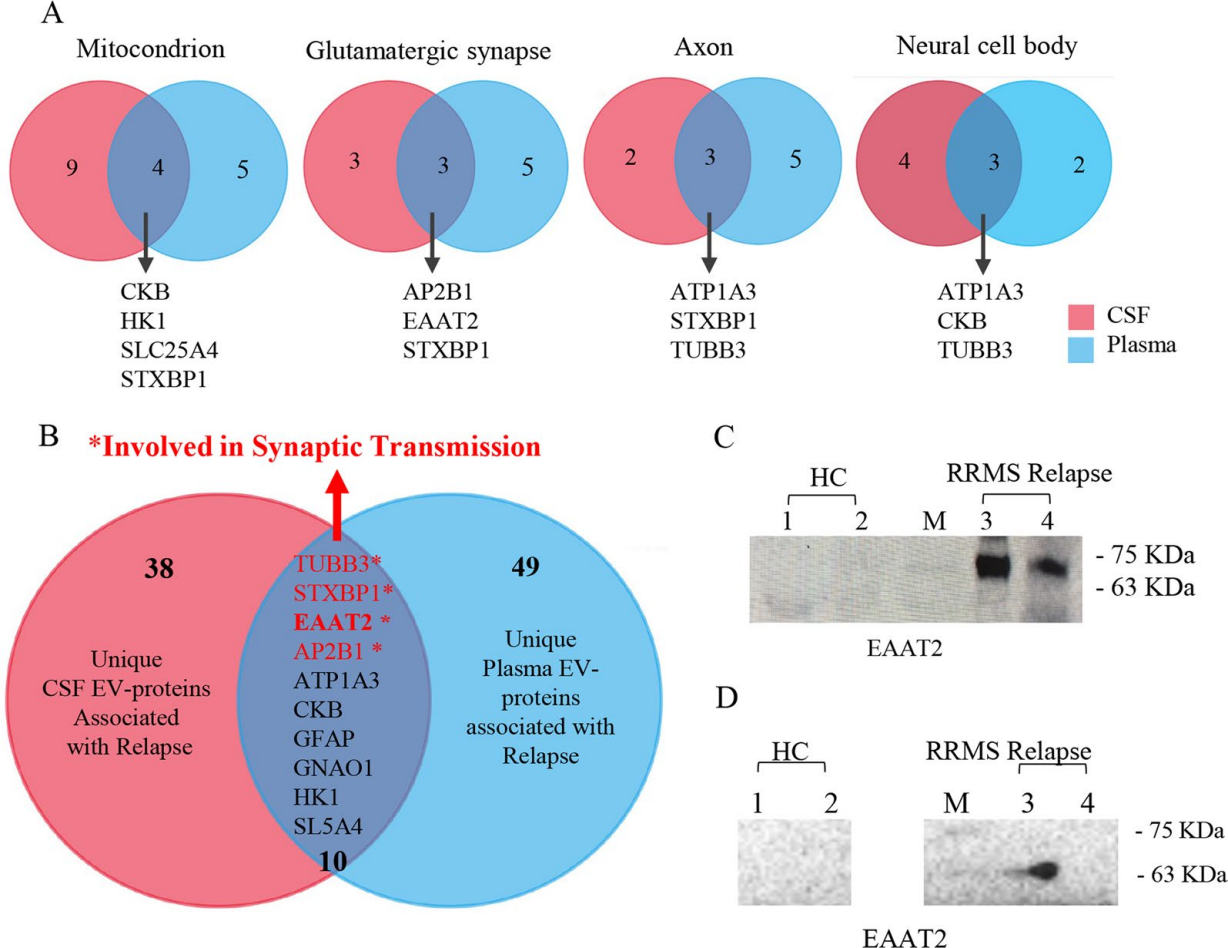
Comparison between CSF and plasma EV proteomes associated with MS relapses. **A** Shared EV proteins belonging to different cellular components. **B** Venn diagram showing ten common proteins, four of which involved in synaptic transmission. **C** Representative Western blot of SEC-purified EVs from plasma samples of relapsing RRMS patients and healthy controls for EAAT2 detection. Lanes 1 and 2: SEC-purified plasma EV samples of 2 healthy controls; Lane: M marker; Lanes 3 and 4: SEC-purified plasma EV samples of 2 relapsing RRMS patients. **D** Representative Western blot of SEC-EVs 20K and f SEC-EVs 100K from plasma samples of a relapsing RRMS patient and a healthy control for EAAT2 detection. Lanes 1 and 2: SEC-EVs 20K and SEC-EVs 100K respectively of a healthy control; Lane: M marker; Lanes 3 and 4: SEC-EVs 20K and f SEC-EVs 100K respectively of a relapsing RRMS patient

We next verified whether CSF EVs showed the same protein signature of plasma-derived EVs associated with MS relapses. Proteomic analysis of SEC-purified CSF EVs from samples of 7 RRMS patients (4 in relapse and 3 in remission), detected a total of 152 proteins (Supplementary Table 5), distributed as reported in Venn diagram (Fig. 4B). Moreover, SEC-purified CSF EVs were analysed by TEM (Fig. 4A). The protein list derived from CSF samples of each group analysed is reported in Supplementary Tables 6 and 7. The matrix charts, showing pairwise comparison of shared EV proteins between subjects of the same group (relapsing and remitting groups), are reported in Supplementary Fig. 2D and 2E. Similar to the results obtained for the plasma EV proteome, FunRich tool for cellular component and Vesiclepedia database showed that the total identified proteins were significantly enriched in extracellular vesicles (*p* ≤ 0.001) (Fig. 4C). Furthermore, as shown in Venn diagram (Fig. 4B), 48 unique proteins were associated with relapse (Table 2). These proteins, classified into the CC GO term, were significantly associated with neuronal cells, synapsis, axon and mitochondrion as well as with thick filaments of sarcomeres, suggesting a potential involvement of striated muscle in MS pathology during relapses (Fig. 4D). Interestingly, as shown for plasma-derived EV proteins associated with MS relapses, also for this set of 48 proteins, synaptic transmission pathway is among the most significantly enriched pathways obtained by Reactome (*p* < 0.001) (Fig. 4E). The comparison between proteins of CSF and plasma EVs associated with relapse (Fig. 5A and 5B), showed ten common proteins (Table 3), four of which are involved in synaptic transmission, namely tubulin β-3 chain, AP-2 complex/b unit β, syntaxin-binding protein 1 and excitatory amino-acid transporter 2 (EAAT2). Among these proteins, EAAT2, found in 66% and 75%, of plasma- and CSF-derived EVs, respectively (Table 3), is the dominant glutamatergic transporter in the CNS which is mainly expressed by astrocytes and involved in glutamate homeostasis dysfunction, a key feature in MS pathogenesis [20, 21]. Taking into account that decreased expression of glutamate transporters on astrocyte surface during neuroinflammation may result in excessive extracellular glutamate and neurotoxicity, we have considered EAAT2 worthy of further investigation as potential MS biomarker.

**Table 3:** Common plasma and CSF EV proteins associated with relapses

	Acc. number	Gene names	Protein name	Plasma relative frequency	CSF relative frequency
1	Q13509	TUBB3, TUBB4	Tubulin β-3 chain	4/6	3/4
2	P12235	SLC25A4, AAC1, ANT1	ADP/ATP translocase 1	5/6	4/4
3	P43004	SLC1A2, EAAT2, GLT1	Excitatory amino acid transporter 2	4/6	3/4
4	P13637	ATP1A3	Sodium/potassium-transporting ATPase/b unit α-3	5/6	3/4
5	P12277	CKB, CKBB	Creatine kinase B-type	1/6	2/4
6	P61764	STXBP1, UNC18A	Syntaxin-binding protein 1	1/6	1/4
7	P14136	GFAP	Glial fibrillary acidic protein	1/6	1/4
8	P09471	GNAO1	Guanine nucleotide-binding protein G(o)/b unit α	1/6	1/4
9	P19367	HK1	Hexokinase-1	1/6	1/4
10	P63010	AP2B1, ADTB2, CLAPB1	AP-2 complex/b unit β	2/6	1/4

The presence of EAAT2 protein in plasma samples of SEC-purified EVs from two relapsing RRMS patients, used for proteomic experiments, was confirmed by western blot analysis (Fig. 5C). Importantly, proteomic analysis performed in separated samples of SEC EV 20K and SEC EV 100K of three relapsing RRMS patients showed that the majority of proteins found associated with relapse, including EAAT2 protein, were detected in SEC EV 20K but not in SEC EV 100K samples, as reported in Venn diagram in Supplementary Fig. 7. This finding was confirmed by western blot analysis detecting EAAT2 protein in SEC EV 20K but not in SEC EV 100K samples (Fig. 5D), encouraging us to investigate the presence of such protein on surface of large EVs ranging in size from 0.5 to 1 µm in flow cytometry [22].

### EAAT2 detection on plasma EV surface

Aiming at detecting EAAT2 protein on plasma EV surface in a larger MS patient cohort, a flow cytometry-based approach, suitable to detect low abundant EV subsets, like CNS-derived EVs, in a complex body fluid such as plasma, was established. The sample processing protocols for EV labelling with currently used fluorescent dyes involve the employment of high-speed centrifugation causing the formation of EV aggregates or morphological changes that may lead to erroneous data interpretation.

In order to ensure reproducibility of the results in flow cytometry, we applied a “no washing” strategy, that does not require isolation or concentration of EVs from plasma samples prior to EV staining and unbounded fluorescent probe removal, after EV labelling. After using a gating strategy for EV detection based on physical parameters (size and complexity) (Supplementary Fig. 4A), a thiol-based fluorescence labelling method (MTG probe), was used [23] to rapidly and accurately identify the EV population in the 0.5–1 µm gate. The evaluation of the efficiency and the specificity of the MTG to bind to EV free-thiol groups and discern between EVs and large lipoproteins in flow cytometry is reported in Supplementary Results.

**Fig. 6:**
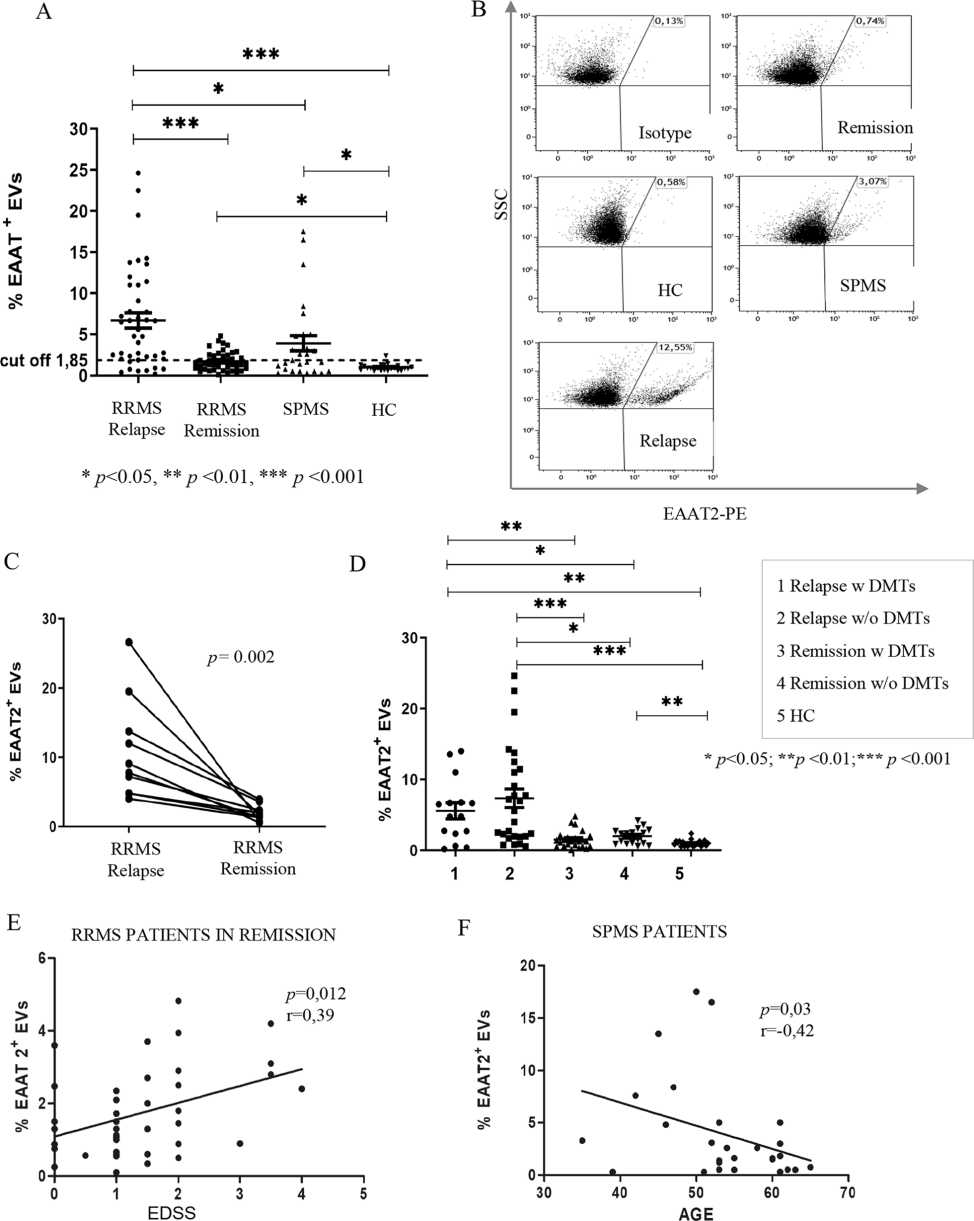
FACS analysis of EAAT2 on EV surface in plasma of MS patients and healthy controls and correlation analysis. **A** EAAT2^**+**^EVs in MTG-positive gate in plasma samples from 42 RRMS patients in relapse, 41 RRMS patients in remission, 27 SPMS patients and 23 healthy subjects. Significance of differences among groups were evaluated by Kruskal–Wallis test. The Mann–Whitney U test was used to compare differences between two independent groups. Cut-off value (mean + 2SD) was calculated. Mean ± standard error of the mean (SEM) values are shown as horizontal lines. **B** Representative dot plots showing the percentage of EAAT2^**+**^EVs in MTG-positive gate for each group considered. Isotype antibody was used as negative control. **C** EAAT2^**+**^EVs in MTG-positive gate in plasma samples longitudinally collected from 10 RRMS patients during relapse and remission. Wilcoxon Signed Rank test for paired samples shows a statistically significant difference (*p* = 0.002) between the two groups (**D**) Comparison of EAAT2^**+**^EV percentage between RRMS patients with or without DMT treatment. Percentage of EAAT2^**+**^EVs in MTG-positive gate in plasma samples of RRMS patients under DMT (15 RRMS patients in relapse and 24 RRMS patients in remission), and RRMS patients without therapy (27 RRMS patients in relapse and 17 RRMS patients in remission). Each dot represents an individual subject. Significance of differences among groups were evaluated by Kruskal–Wallis test. The Mann–Whitney U test was used to compare differences between two independent groups. Mean ± standard error of the mean (SEM) values are shown as horizontal lines. (*p < 0.05, **p < 0.01, ***p < 0.001). **E** Significant positive correlation between plasma EAAT2+EV percentage values and EDSS score in remitting RRMS patients. **F** Significant inverse correlation between plasma EAAT2+EV percentage values and age in SPMS patients. Correlations were determined by Spearman's Rho using GraphPad Prism 5 software.

The presence of EVs carrying EAAT2 protein (EV-EAAT2) on their surface was assessed in plasma samples of 110 MS patients (42 RRMS patients in relapse, 41 RRMS patients in remission, 27 SPMS patients) and 23 healthy controls. The flow cytometry results, showing the percentages of EV-EAAT2 in the total plasma EVs, are presented in Fig. 6A and 6B, while the related descriptive statistics are reported in Table 4. Non-parametric Kruskal–Wallis test revealed statistically significant differences (*p* < 0.001) among the four groups and the Mann–Whitney U test showed a statistically significant increase in the percentage of plasma EV-EAAT2 in relapsing RRMS patients compared to remitting RRMS patients, SPMS patients and healthy controls (Fig. 6A). Moreover, a statistically significant increase in the percentage of plasma EV-EAAT2 in remitting RRMS patients and SPMS patients versus healthy controls was observed. These findings suggest that, although plasma EV-EAAT2 levels are significantly increased in all clinical MS forms respect to healthy controls, the highest EV-EAAT2 levels are observed in relapsing RRMS patients.

**Table 4:** Plasma EV- EEAT2 in different MS clinical phases

	RRMS Relapse	RRMS Remission	SPMS	HC	Total	*p*-value^a^
*Plasma EV-EEAT2 (%)*						***p* < 0.001**
No. of patients	42	41	27	23	133	
Mean (%)	6,7	1,7	3,9	1	3,6	
Std. Deviation	6,1	1,2	4,831	0,4	4,7	
Std. Error	0,94	0,2	0,9	0,08	0,4	
Range	0,2/24,6	0,1/4,8	0,3/17,5	0,57/2,4	0,1/24,6	
*EEAT2 correlation (r/p value)*						
Age	0,19/0,22	0,15/0,36	− 0,42/0,03*	0,04/0,86	–	
EDSS score	− 0,4/0,79	0,39/0,012*	− 0,76/0,71		–	
Disease Duration	0,17/0,27	− 0,05/0,73	0,08/0,67	–	–	

^a^Kruskal–Wallis test; the statistical significance are in bold

^*^Rho of Spearman’s test; the statistical significance are in bold

To explore whether changes in the percentage of plasma EV-EAAT2 are related to different MS phases over time, the presence of EV-EAAT2 was evaluated in paired relapse/remission plasma samples collected from 10 RRMS patients within 12 months of the first plasma sampling (Fig. 6C). Wilcoxon Signed Rank test for paired samples showed a significant difference between EV-EAAT2 percentages in relapse and remission (*p* = 0.002), confirming the association of highest plasma EV-EAAT2 levels with MS relapses. In order to investigate the effect of drug treatment on plasma EV- EAAT2 frequency, flow cytometry results were analysed taking into account whether RRMS patients received DMTs or were untreated, since at least 3 months. Kruskal–Wallis test showed a statistically significant differences among all groups analysed (*p* < 0.001) (Table 5). The Mann–Whitney U test revealed that plasma EV-EAAT2 percentages were significantly increase in DMT-treated relapsing patients compared to both DMT-treated and untreated remitting patients (*p* = 0.0026 and *p* = 0.023 respectively) and healthy controls (*p* = 0.0014), and in untreated relapsing patients compared to both DMT-treated and untreated remitting patients (*p* < 0.0001 and *p* = 0.0114 respectively) and healthy controls (*p* < 0.0001) (Fig. 6D). Interestingly, plasma EV-EAAT2 percentages were significantly increased in untreated but not in DMT-treated remitting patients versus healthy controls (*p* = 0.0027). These findings showed that relapsing RRMS patients irrespective of exposure to DMT showed higher plasma EV-EAAT2 levels than DMT-treated and untreated remitting patients and healthy controls (Fig. 6D and Table 5).

**Table 5:** Plasma EV-EAAT2 in RRMS patients

	Relapsing RRMS patients		Remission RRMS patients		HC	Total	*p-*value^a^
	With DMT	Without DMT	With DMT	Without DMT			
*Plasma EV- EEAT2 (%)*							***p* < 0.001**
N	15	27	24	17	23	106	
Mean	5,6	7,3	1,5	2	1	3,5	
Std. Deviation	4,4	6,9	1,2	1,1	0,4	4,6	
Std. Error	1,1	1,3	0,2	0,3	0,08	0,4	
Range	0,2–14	0,5–24,6	0,1–4,8	0,6–4,2	0,6–2.4	0,1–24,6	

^a^Kruskal–Wallis test; The statistical significance are in bold

For each patient group, there were no statistically significant correlations between plasma EV-EAAT2 levels and sex (Supplementary Table 8), or disease duration (Table 4). Meanwhile, plasma EV-EAAT2 levels of RRMS patients in remission correlated positively with the EDSS score (*r* = 0.39/*p* = 0.012) (Table 4 and Fig. 6E). Moreover, it has been observed a significant negative correlation between plasma EV-EAAT2 levels and age in SPMS patients (*r* = − 0.42/*p* = 0.03) (Table 4 and Fig. 6F).

To verify whether astrocyte-derived EVs expressed EAAT2 protein on their surface, we used the U251 multiform glioblastoma cell line, which expresses TLRs and TNF receptor 1, as an astrocyte-like model responding to inflammatory stimuli [24, 25]. Flow cytometry analysis detected EAAT2 protein on the EV surface obtained from the culture supernatant of U251 cells; EAAT2 EV levels were similar in untreated cells and in cells treated with different inflammatory stimuli, like LPS, TNF-α and serum starvation (Supplementary Fig. 6). This finding was confirmed by Western blot analysis, showing the presence of EAAT2 protein in U251-derived EVs isolated from culture supernatant of untreated and serum starved cell line, but not in K562-derived EVs, used as negative control (data not shown).

## Discussion

This study is the first to investigate the proteome of plasma and CSF EVs from RRMS patients aiming at identifying candidate MS biomarkers using a proteomic profiling comparison approach [26–28].

The main finding is that a set of proteins detected in both plasma and CSF EVs were associated with MS relapses and were significantly enriched in proteins involved in synaptic transmission, which is known to be dysregulated in MS [29]. Interestingly, plasma EVs associated with MS relapses carry several proteins derived from CNS cells, particularly proteins expressed in the synapse, axon and myelin sheet. These findings confirm the release of CNS-derived EVs into the peripheral blood and the power of the strategy adopted here to identify novel candidate disease biomarkers.

Although growing evidence showed that small EV can cross BBB in bidirectional way under physiological and pathological conditions [13, 30], we found also large EVs, carrying the major CNS proteins associated with relapse phase, in periphery, suggesting their ability to cross altered BBB during neuroinflammation.

Among neuronal proteins found in plasma EVs, particularly interesting are synaptotagmin1 (SYT1), syntaxin binding protein 1(STXBP1), synaptosome associated protein 25(SNAP25) and vesicle associated membrane protein 2(VAMP2). These proteins are distributed along the axon and form the SNARE complex which is critical for synaptic vesicle fusion and neurotransmitter release [31]. Other proteins found in plasma EVs and expressed in neurons are neurofilament medium and light chains and ATPase Na+/K+ transporting subunit α 3 (ATP1A3), an enzyme involved in the action potential propagation during neuronal depolarization [32]. Not only neuronal, but also glial proteins, like GFAP, [33] EAAT2, glutamine synthetase (GLNA), and the major CNS myelin protein, proteolipid protein 1 (PLP1) were detected in plasma EVs. Moreover, we identified mitochondrial proteins involved in the traffic of various solutes across the inner mitochondrial membrane (SLC25A4 and SLC25A6), glucose metabolism (HXK1) and oxidative phosphorylation (ATP5F1A and NDUFS1). Circulating EVs carrying mitochondrial components, classified as mitovesicles, may reflect mitochondrial dysfunction which is thought to have a key role in MS pathogenesis [34, 35].

Proteomic analysis of CSF EVs associated with MS relapses revealed the presence of proteins derived from the same CNS cellular components found in plasma EVs, such as synapse, axon and mitochondria. Otherwise, proteomic analysis of CSF EVs also detected proteins expressed in peripheral tissues, such as components of the sarcomere (ARF3, ACTA1, MYH2, MYH3, MYH7 and MYH8), thereby confirming the bidirectional EV trafficking between the CNS and the periphery. The presence of circulating EVs carrying sarcomeric proteins might indicate skeletal muscle damage, probably triggered by circulating pro-inflammatory mediators during MS relapses. This suggestion is supported by the observation that histological and molecular changes in skeletal muscle linked to mitochondrial dysfunction occur at disease onset in EAE, a widely used animal model of MS [36, 37]. In contrast, during disease progression, major changes in the muscle structure leading to motor deficits, could be attributed to impaired axonal conduction resulting from chronic demyelination [38].

Of major relevance for a better understanding of MS pathogenesis is the presence of circulating glia-derived EVs during relapses that may shed further light on the link between neuroinflammation and synaptic dysfunction in MS pathology.

During disease exacerbation, studies performed in the EAE model and in MS patients using transcranial magneting stimulation techniques indicate that immune-mediated inflammation is associated not only with CNS demyelination but also with altered synaptic transmission [39–41]. Specifically, neuroinflammation induces an increase of excitatory glutamatergic transmission, a decrease in inhibitory GABAergic transmission, an altered glutamate uptake by astrocytes and a loss of synapses, all of which contribute to diffuse synaptopathy [40, 41].

Glutamatergic synapse dysfunction, caused by an excessive activation of the ionotropic NMDA receptors of glutamate, which can be also produced by inflammatory cells, including activated microglia, as well as reduced glutamate uptake in the synaptic cleft, can lead to excitotoxicity and synaptic loss. Impaired or decreased expression of high-affinity sodium-dependent glutamate transporters, EAATs, particularly EAAT2/GLT1 responsible for the majority of the glutamate uptake in CNS, makes neurons and oligodendrocytes highly susceptible to excitotoxicity [21, 42].

Studies in EAE models and MS brain lesions, have shown that EAATs, including EAAT2, [21, 43, 44] are reduced in CNS, predominantly in astrocyte, and that EAAT downregulation induced by inflammatory stimuli, such as interleukin 1β and TNFα, is associated with altered glutamate uptake [45, 46].

Experiments in cultured rat astrocytes and the present results in the U251 multiform glioblastoma cell line show that EAAT2 is incorporated in EVs, under physiologic and inflammatory conditions.

The evidence of EAATs carried by EVs released from spinal explants after nerve injury to have the ability to uptake extracellular glutamate, along with our proteomic detection of GLNA, responsible for conversion of glutamate to glutamine, suggest that glia-derived EVs, carrying proteins involved in synaptic glutamate clearance, may have a key role in maintaining glutamate homeostasis during neuroinflammation [47].

Owing to its central role in preventing excitotoxicity and its presence in both CSF and plasma EVs during MS relapses, EAAT2 was selected for validation as biomarker of disease activity in RRMS and evaluation in SPMS patients.

To this end, we developed a strategy allowing for rapid flow cytometric detection of EAAT2 on plasma EV surface using an efficient and specific fluorescent probe (MTG) that allowed to identify EVs without the need for a purification step before and after antibody labelling.

Screening of plasma samples for the presence of EV-EAAT2 showed a statistically significant increase in EV-EAAT2 levels in relapsing RRMS patients compared to remitting RRMS patients, SPMS patients and healthy controls, regardless of DMT exposure. Further experiments in paired relapse/remission plasma samples collected from RRMS patients, highlighted changes of EV-EAAT2 level associated with different phases of the disease overtime and confirmed the association of highest plasma EV-EAAT2 level with MS relapses. Interestingly, when remitting RRMS patients were subdivided in untreated or DMT-treated patient groups, only the untreated group showed a significant increase of plasma EV-EAAT2 levels respect to healthy controls.

Despite of the small number of samples analysed, our study also provides preliminary evidence of a positive correlation of plasma EV-EAAT2 levels with EDSS score in RRMS during remission and of a negative correlation with age in SPMS patients.

There are some limitations related to the current study. A downside of the proteomic approach is that highly abundant proteins can mask the detection of low abundance proteins, especially when EVs purified from plasma samples are analysed. Furthermore, in each subject, plasma EVs, originated from different body districts, with a large diversity of proteins, could differ in their number and protein content.

In view of these considerations, the comparison between CSF and plasma EV proteomes associated with MS relapses showed only ten common proteins, although the most of the proteins identified in both proteomes were significantly associated with neuronal cells, synapsis, axon and mitochondrion. Furthermore, sarcomeric proteins were detected in CSF but not in plasma EVs otherwise myelin proteins were found in plasma but not in CSF EVs. Finally, the lack of commercial assays and the difficulty of developing specific assays for detecting proteins carried by EVs prevented us from analysing other EV proteins associated with relapses as potential MS biomarkers. Despite these limitations, the present study highlights EAAT2 as a candidate biomarker for MS relapses.

## Conclusion

Our strategy based on comparison of proteomic signatures of CSF and plasma EVs, purified from samples of RRMS patients, turned out to be appropriate not only for supplying novel biomarkers of MS disease activity, but also to improve the knowledge about the pathological mechanisms underlying the disease. Indeed, the proteomic analysis of both CSF and plasma EV profiles associated with MS relapses revealed several proteins involved in synaptic transmission, which is known to be altered in MS during neuroinflammation.

Chosen among ten shared proteins between CSF and plasma EV proteomes associated with MS relapses, EAAT2 on plasma EV surface detected by using a novel and highly reproducible flow cytometry-based approach, showed to be a promising biomarker of MS relapses. Additionally, the plasma EV-EAAT2 levels positively correlated with EDSS score in RRMS during remission and negatively correlated with age in SPMS patients, suggesting the need for more research to evaluate plasma EV-EAAT2 also as potential prognostic biomarker for PIRA and the transition from RRMS towards SPMS [48, 49].

Moreover, the development of an easy-to use quantitative immunoassay to measure plasma EV-EAAT2 with high sensitivity and specificity would be helpful to evaluate its usefulness in the clinical practice.

### Supplementary Information

Additional file 1.

Additional file 2.

Additional file 3.

Additional file 4.

Additional file 5.

Additional file 6.

Additional file 7.

Additional file 8.

Additional file 9.

Additional file 10.

Additional file 11.

Additional file 12.

Additional file 13.

Additional file 14.

Additional file 15.

Additional file 16.

Additional file 17.

## Data Availability

The proteomic data have been deposited to the ProteomeXchange Consortium via the PRIDE partner repository with the following link: http://massive.ucsd.edu/ProteoSAFe/status.jsp?task=78fca3bcbfa54f8784ea45ec4282c670

## Abbreviations

DMT: Disease-modifying therapy
EAAT2: Excitatory amino-acid transporter 2
EDSS: Expanded Disability Status Scale
EVs: Extracellular vesicles
GFAP: Glial fibrillary acidic protein
MTG: MITO Tracker Green FM
MS: Multiple sclerosis
sNfl: Serum Neurofilament-light chain
PPP: Platelet-poor plasma
PIRA: Progression independent of relapse activity
RRMS: Relapsing remitting MS
SEC: Size-exclusion chromatography
SPMS: Secondary progressive MS
TEM: Transmission electron microscopy

## References

[1] Scalfari A, Neuhaus A, Degenhardt A, et al. The natural history of multiple sclerosis: a geographically based study 10: relapses and long-term disability. Brain. 2010;133:1914–29. 10.1093/brain/awq118. 20534650 10.1093/brain/awq118 PMC2892939

[2] Chard D, Trip SA. Resolving the clinico-radiological paradox in multiple sclerosis. F1000Res. 2017;6:1828. 10.12688/f1000research.11932.1. 29093810 10.12688/f1000research.11932.1 PMC5645703

[3] Hickman SJ. Optic nerve imaging in multiple sclerosis. J Neuroimaging. 2007;17:42S-45S. 10.1111/j.1552-6569.2007.00136.x. 17425734 10.1111/j.1552-6569.2007.00136.x

[4] Kearney H, Miller DH, Ciccarelli O. Spinal cord MRI in multiple sclerosis–diagnostic, prognostic and clinical value. Nat Rev Neurol. 2015;11:327–38. 10.1038/nrneurol.2015.80. 26009002 10.1038/nrneurol.2015.80

[5] Mills EA, Mirza AY, Mao-Draayer Y. Emerging approaches for validating and managing multiple sclerosis relapse. Front Neurol. 2017;8:116. 10.3389/fneur.2017.00116. 28424654 10.3389/fneur.2017.00116 PMC5372802

[6] Cook SD, Dhib-Jalbut S, Dowling P, et al. Use of magnetic resonance imaging as well as clinical disease activity in the clinical classification of multiple sclerosis and assessment of its course. Int J MS Care. 2012;14:105–14. 10.7224/1537-2073-14.3.105. 24453741 10.7224/1537-2073-14.3.105 PMC3882992

[7] Koch-Henriksen N, Koch-Henriksen N, Sørensen PS, Magyari M. Relapses add to permanent disability in relapsing multiple sclerosis patients. Multiple Sclerosis Relat Disord. 2021;53: 103029. 10.1016/j.msard.2021.103029. 10.1016/j.msard.2021.103029 34116481

[8] Lublin FD, Häring DA, Ganjgahi H, et al. How patients with multiple sclerosis acquire disability. Brain. 2022;145:3147–61. 10.1093/brain/awac016. 35104840 10.1093/brain/awac016 PMC9536294

[9] Barro C, Benkert P, Disanto G, et al. Serum neurofilament as a predictor of disease worsening and brain and spinal cord atrophy in multiple sclerosis. Brain. 2018;141:2382–91. 10.1093/brain/awy154. 29860296 10.1093/brain/awy154

[10] Meier S, Willemse EAJ, Schaedelin S, et al. Serum glial fibrillary acidic protein compared with neurofilament light chain as a biomarker for disease progression in multiple sclerosis. JAMA Neurol. 2023;80:287–97. 10.1001/jamaneurol.2022.5250. 36745446 10.1001/jamaneurol.2022.5250 PMC10011932

[11] Koini M, Pirpamer L, Hofer E, et al. Factors influencing serum neurofilament light chain levels in normal aging. Aging. 2021;13:25729–38. 10.18632/aging.203790. 34923481 10.18632/aging.203790 PMC8751593

[12] Zamboni S, D’Ambrosio A, Margutti P. Extracellular vesicles as contributors in the pathogenesis of multiple sclerosis. Mult Scler Relat Disord. 2023;71: 104554. 10.1016/j.msard.2023.104554. 36842311 10.1016/j.msard.2023.104554

[13] García-Romero N, Carrión-Navarro J, Esteban-Rubio S, et al. DNA sequences within glioma-derived extracellular vesicles can cross the intact blood-brain barrier and be detected in peripheral blood of patients. Oncotarget. 2017;8:1416–28. 10.18632/oncotarget.13635. 27902458 10.18632/oncotarget.13635 PMC5352065

[14] Thompson AJ, Banwell B, Barkhof F, et al. Diagnosis of multiple sclerosis: 2017 revisions of the McDonald criteria. Lancet Neurol. 2018;17:162–73. 10.1016/S1474-4422(17)30470-2. 29275977 10.1016/S1474-4422(17)30470-2

[15] Coumans FAW, Brisson AR, Buzas EI, et al. Methodological guidelines to study extracellular vesicles. Circ Res. 2017;120(10):1632–48. 10.1161/CIRCRESAHA.117.309417. 28495994 10.1161/CIRCRESAHA.117.309417

[16] Puthenparampil M, Miante S, Federle L, et al. BAFF is decreased in the cerebrospinal fluid of multiple sclerosis at clinical onset. J Neuroimmunol. 2016;297:63–7. 10.1016/j.jneuroim.2016.05.013. 27397077 10.1016/j.jneuroim.2016.05.013

[17] Lalle M, Camerini S, Cecchetti S, Sayadi A, Crescenzi M, Pozio E. Interaction network of the 14–3-3 protein in the ancient protozoan parasite *Giardia duodenalis*. J Proteome Res. 2012;11:2666–83. 10.1021/pr3000199. 22452640 10.1021/pr3000199

[18] Simeone P, Celia C, Bologna G, et al. Diameters and fluorescence calibration for extracellular vesicle analyses by flow cytometry. Int J Mol Sci. 2020;21:7885. 10.3390/ijms21217885. 33114229 10.3390/ijms21217885 PMC7660682

[19] Calogero A, Lombari V, De Gregorio G, et al. Inhibition of cell growth by EGR-1 in human primary cultures from malignant glioma. Cancer Cell Int. 2004;4:1. 10.1186/1475-2867-4-1. 14711380 10.1186/1475-2867-4-1 PMC324562

[20] Kostic M, Zivkovic N, Stojanovic I. Multiple sclerosis and glutamate excitotoxicity. Rev Neurosci. 2013;24:71–88. 10.1515/revneuro-2012-0062. 23152401 10.1515/revneuro-2012-0062

[21] Werner P, Pitt D, Raine CS. Multiple sclerosis: altered glutamate homeostasis in lesions correlates with oligodendrocyte and axonal damage. Ann Neurol. 2001;50:169–80. 10.1002/ana.1077. 11506399 10.1002/ana.1077

[22] Sachetto ATA, Archibald SJ, Hisada H, et al. Tissue factor activity of small and large extracellular vesicles in different diseases. Res Pract Thromb Haemost. 2023;7(3): 100124. 10.1016/j.rpth.2023.100124). 37012986 10.1016/j.rpth.2023.100124) PMC10015082

[23] Roberts-Dalton HD, Cocks A, Falcon-Perez JM, et al. Fluorescence labelling of extracellular vesicles using a novel thiol-based strategy for quantitative analysis of cellular delivery and intracellular traffic. Nanoscale. 2017;9:13693–706. 10.1039/c7nr04128d. 28880029 10.1039/c7nr04128d

[24] Zeuner MT, Vallance T, Vaiyapuri S, Cottrell GS, Widera D. Development and characterisation of a novel NF- κ B reporter cell line for investigation of neuroinflammation. Mediators Inflamm. 2017;2017:6209865. 10.1155/2017/6209865. 28790798 10.1155/2017/6209865 PMC5534271

[25] Chakraborty S, Li L, Tang H, et al. Cytoplasmic TRADD confers a worse prognosis in glioblastoma. Neoplasia. 2013;15:888–97. 10.1593/neo.13608. 23908590 10.1593/neo.13608 PMC3730041

[26] Pieragostino D, Lanuti P, Cicalini I, et al. Proteomics characterization of extracellular vesicles sorted by flow cytometry reveals a disease-specific molecular cross-talk from cerebrospinal fluid and tears in multiple sclerosis. J Proteomics. 2019;204: 103403. 10.1016/j.jprot.2019.103403. 31170500 10.1016/j.jprot.2019.103403

[27] Jeannin P, Chaze T, Gianetto QG, et al. Proteomic analysis of plasma extracellular vesicles reveals mitochondrial stress upon HTLV-1 infection. Sci Rep. 2018;8:5170. 10.1038/s41598-018-23505-0. 29581472 10.1038/s41598-018-23505-0 PMC5980083

[28] Muraoka S, Jedrychowski MP, Yanamandra K, et al. Proteomic profiling of extracellular vesicles derived from cerebrospinal fluid of Alzheimer’s disease patients: a pilot study. Cells. 2020;9:1959. 10.3390/cells9091959. 32854315 10.3390/cells9091959 PMC7565882

[29] Möck EEA, Honkonen E, Airas L. Synaptic loss in multiple sclerosis: a systematic review of human post-mortem studies. Front Neurol. 2021;12: 782599. 10.3389/fneur.2021.782599. 34912290 10.3389/fneur.2021.782599 PMC8666414

[30] Banks WA, Sharma P, Bullock KM, et al. Transport of extracellular vesicles across the blood-brain barrier: brain pharmacokinetics and effects of inflammation. Int J Mol Sci. 2020;21(12):4407. 10.3390/ijms21124407. 32575812 10.3390/ijms21124407 PMC7352415

[31] Wu Z, Dharan N, McDargh ZA, Thiyagarajan S, O’Shaughnessy B, Karatekin E. The neuronal calcium sensor Synaptotagmin-1 and SNARE proteins cooperate to dilate fusion pores. Elife. 2021;10: e68215. 10.7554/eLife.68215. 34190041 10.7554/eLife.68215 PMC8294851

[32] Zhang X, Lee W, Bian JS. Recent advances in the study of Na+/K+-ATPase in neurodegenerative diseases. Cells. 2022;11:4075. 10.3390/cells11244075. 36552839 10.3390/cells11244075 PMC9777075

[33] Abdelhak A, Foschi M, Abu-Rumeileh S, et al. Blood GFAP as an emerging biomarker in brain and spinal cord disorders. Nat Rev Neurol. 2022;18:158–72. 10.1038/s41582-021-00616-3. 35115728 10.1038/s41582-021-00616-3

[34] Barcelos IP, Troxell RM, Graves JS. Mitochondrial dysfunction and multiple sclerosis. Biology. 2019;8:37. 10.3390/biology8020037. 31083577 10.3390/biology8020037 PMC6627385

[35] Nikić I, Merkler D, Sorbara C, et al. A reversible form of axon damage in experimental autoimmune encephalomyelitis and multiple sclerosis. Nat Med. 2011;17:495–9. 10.1038/nm.2324). 21441916 10.1038/nm.2324)

[36] Park S, Nozaki K, Guyton MK, Smith JA, Ray SK, Banik NL. Calpain inhibition attenuated morphological and molecular changes in skeletal muscle of experimental allergic encephalomyelitis rats. J Neurosci Res. 2012;90:2134–45. 10.1002/jnr.23096. 22715087 10.1002/jnr.23096 PMC12010168

[37] Luque E, Ruz-Caracuel I, Medina FJ, et al. Skeletal muscle findings in experimental autoimmune encephalomyelitis. Pathol Res Pract. 2015;211:493–504. 10.1016/j.prp.2015.02.004. 25769878 10.1016/j.prp.2015.02.004

[38] Neamtu MC, Neamtu OM, Rusu MR, Marin MI, Rusu L. Functional muscle balance assessment in multiple sclerosis. J Back Musculoskelet Rehabil. 2020;33:607–12. 10.3233/BMR-191518. 31743986 10.3233/BMR-191518

[39] Bellingacci L, Mancini A, Gaetani L, Tozzi A, Parnetti L, Di Filippo M. Synaptic dysfunction in multiple sclerosis: a red thread from inflammation to network disconnection. Int J Mol Sci. 2021;22:9753. 10.3390/ijms22189753. 34575917 10.3390/ijms22189753 PMC8469646

[40] Stampanoni Bassi M, Mori F, Buttari F, et al. Neurophysiology of synaptic functioning in multiple sclerosis. Clin Neurophysiol. 2017;128:1148–57. 10.1016/j.clinph.2017.04.006. 28511127 10.1016/j.clinph.2017.04.006

[41] Mandolesi G, Gentile A, Musella A, et al. Synaptopathy connects inflammation and neurodegeneration in multiple sclerosis. Nat Rev Neurol. 2015;11:711–24. 10.1038/nrneurol.2015.222. 26585978 10.1038/nrneurol.2015.222

[42] Coyle JT, Puttfarcken P. Oxidative stress, glutamate, and neurodegenerative disorders. Science. 1993;262:689–95. 10.1126/science.7901908. 7901908 10.1126/science.7901908

[43] Mandolesi G, Musella A, Gentile A, et al. Interleukin-1β alters glutamate transmission at Purkinje cell synapses in a mouse model of multiple sclerosis. J Neurosci. 2013;33:12105–21. 10.1523/JNEUROSCI.5369-12.2013. 23864696 10.1523/JNEUROSCI.5369-12.2013 PMC6794065

[44] Vercellino M, Merola A, Piacentino C, et al. Altered glutamate reuptake in relapsing-remitting and secondary progressive multiple sclerosis cortex: correlation with microglia infiltration, demyelination, and neuronal and synaptic damage. J Neuropathol Exp Neurol. 2007;66:732–9. 10.1097/nen.0b013e31812571b0. 17882017 10.1097/nen.0b013e31812571b0

[45] Ohgoh M, Hanada T, Smith T, et al. Altered expression of glutamate transporters in experimental autoimmune encephalomyelitis. J Neuroimmunol. 2002;125:170–8. 10.1016/s0165-5728(02)00029-2. 11960654 10.1016/s0165-5728(02)00029-2

[46] Loría F, Petrosino S, Hernangómez M, et al. An endocannabinoid tone limits excitotoxicity in vitro and in a model of multiple sclerosis. Neurobiol Dis. 2010;37:166–76. 10.1016/j.nbd.2009.09.020. 19815071 10.1016/j.nbd.2009.09.020

[47] Gosselin RD, Meylan P, Decosterd I. Extracellular microvesicles from astrocytes contain functional glutamate transporters: regulation by protein kinase C and cell activation. Front Cell Neurosci. 2013;7:251. 10.3389/fncel.2013.00251. 24368897 10.3389/fncel.2013.00251 PMC3857901

[48] Tur C, Carbonell-Mirabent P, Cobo-Calvo Á, et al. Association of early progression independent of relapse activity with long-term disability after a first demyelinating event in multiple sclerosis. JAMA Neurol. 2023;80:151–60. 10.1001/jamaneurol.2022.4655. 36534392 10.1001/jamaneurol.2022.4655 PMC9856884

[49] Kleiter I, Ayzenberg I, Havla J, et al. The transitional phase of multiple sclerosis: characterization and conceptual framework. Mult Scler Relat Disord. 2020;44: 102242. 10.1016/j.msard.2020.102242. 32535501 10.1016/j.msard.2020.102242

