# Supplementary figures and images for: Proteomic profile of extracellular vesicles from plasma and CSF of multiple sclerosis patients reveals disease activity-associated EAAT2
Source: J Neuroinflammation. 2024 Sep 2;21:217. doi: 10.1186/s12974-024-03148-x (PMC11370133; doi:10.1186/s12974-024-03148-x)

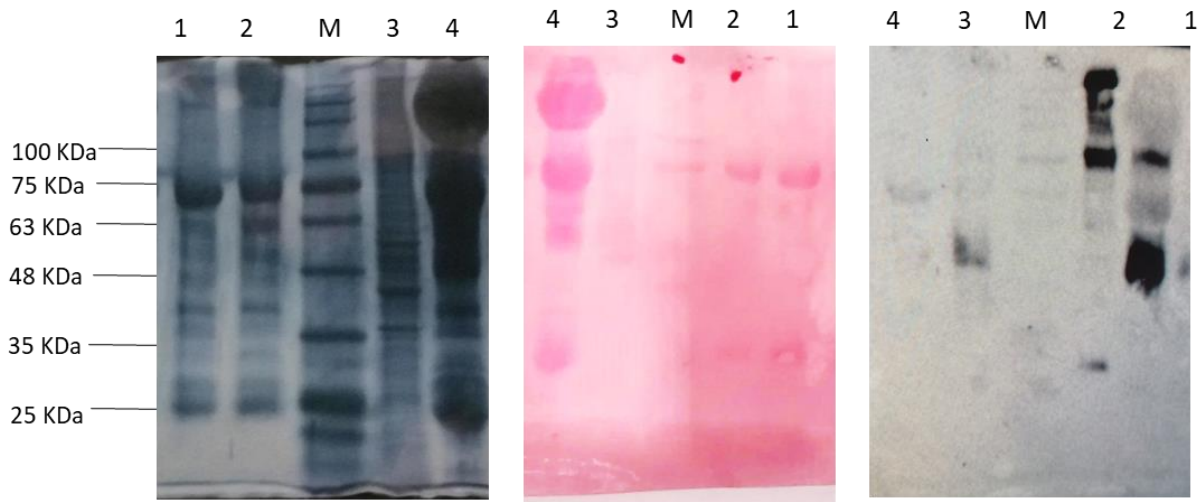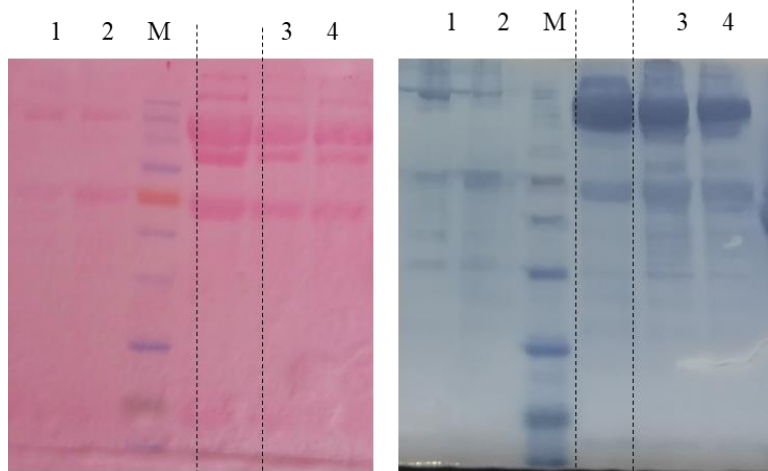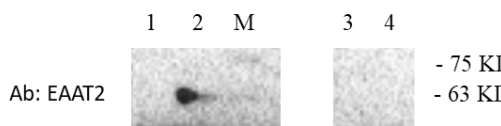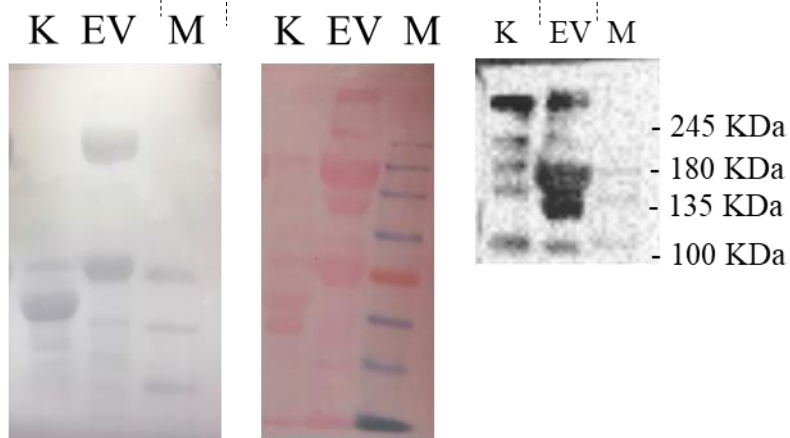

Ab: ApoB48

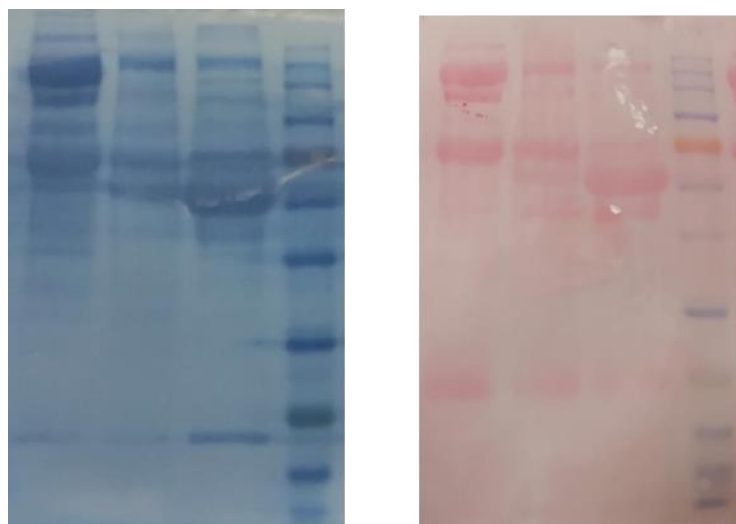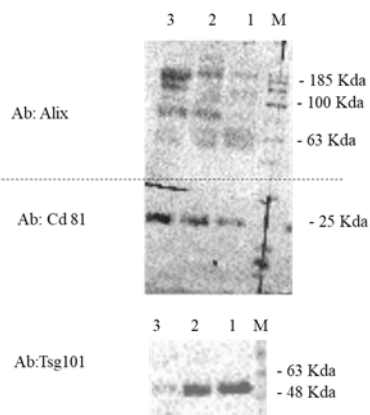

Supplement: Supplementary file 1 — Additional file 1. [file 12974_2024_3148_MOESM1_ESM.pdf]

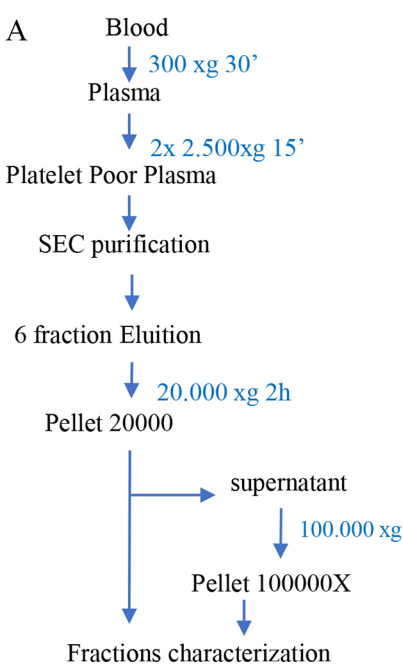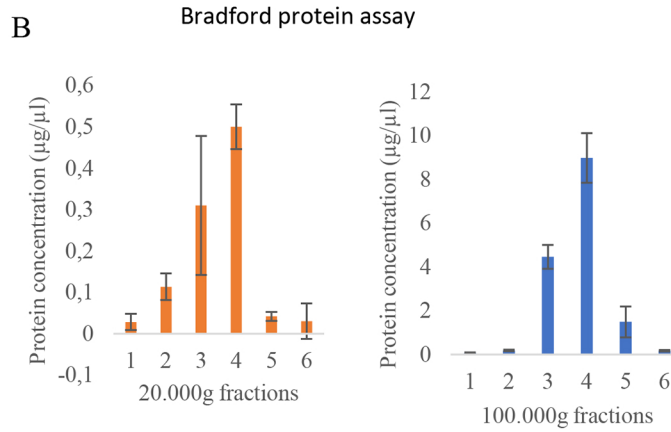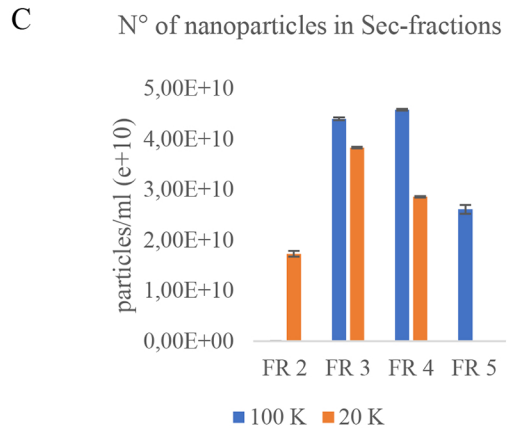

**D** Nanoparticle Tracking Analysis 20.000g Fractions

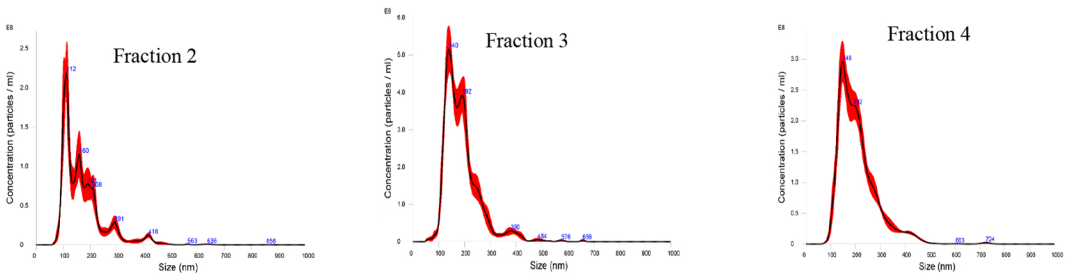

Nanoparticle Tracking Analysis 100.000g Fractions

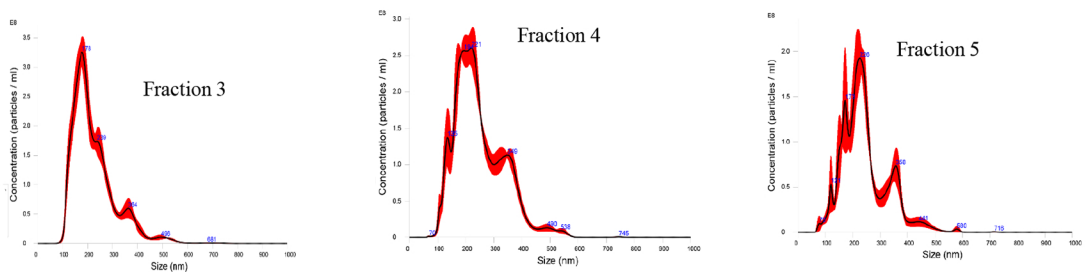

Supplement: Supplementary file 3 — Additional file 3. [file 12974_2024_3148_MOESM3_ESM.pdf]

A

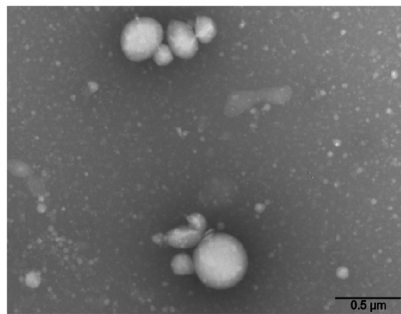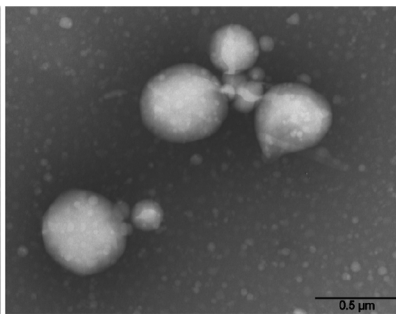

B

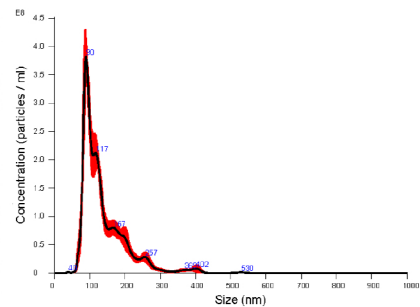

C

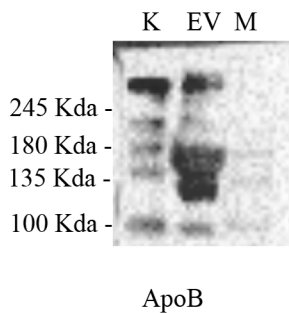

D

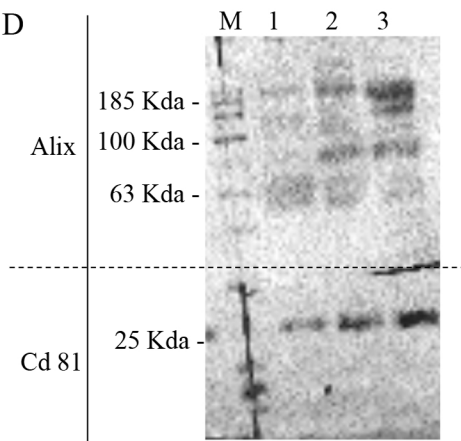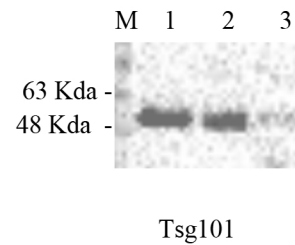

Supplement: Supplementary file 5 — Additional file 5. [file 12974_2024_3148_MOESM5_ESM.pdf]

A

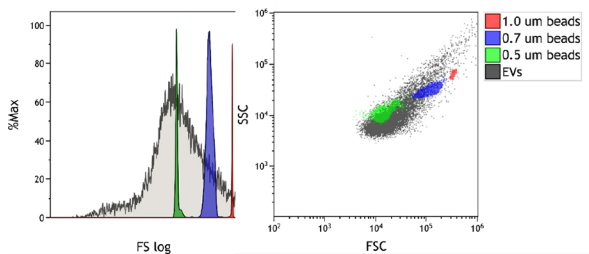

B

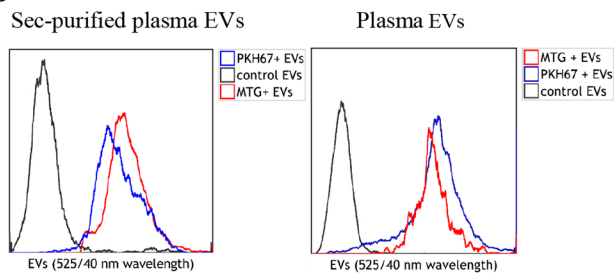

C

### SEC-purified Plasma EVs

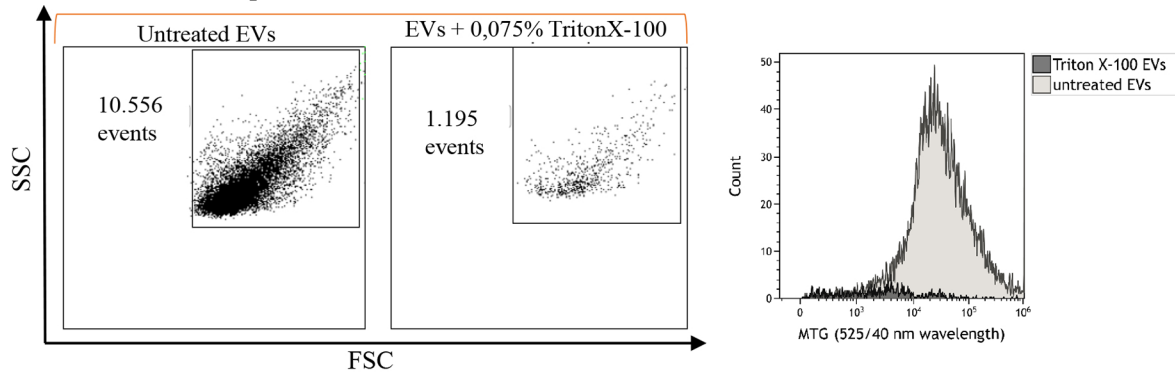

D

### Not purified Plasma EVs

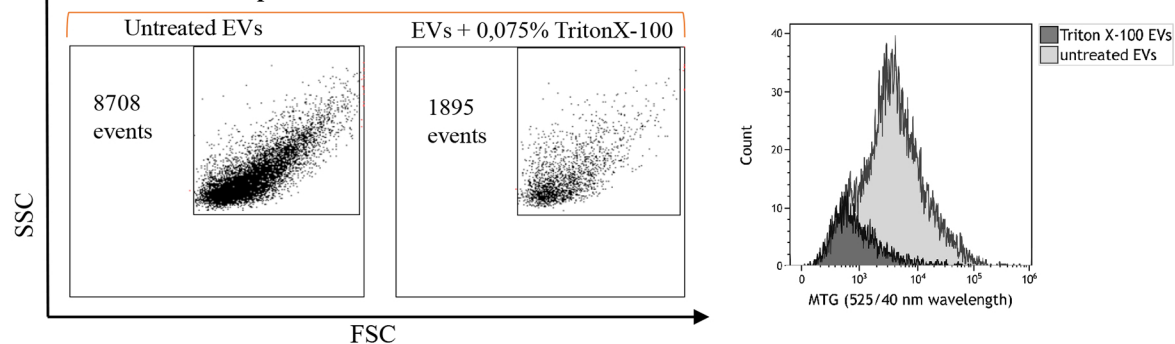

E

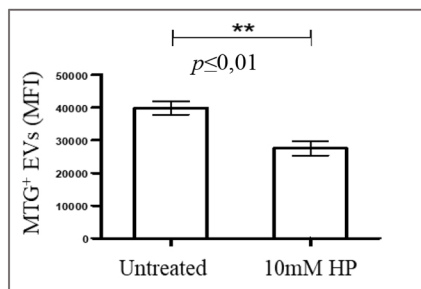

Supplement: Supplementary file 6 — Additional file 6. [file 12974_2024_3148_MOESM6_ESM.pdf]

# SEC-purified plasma EVs

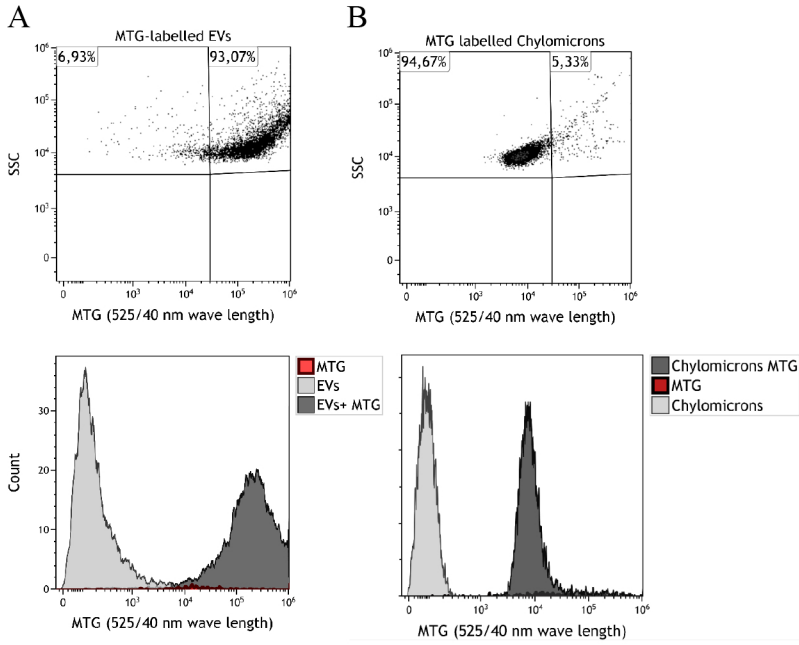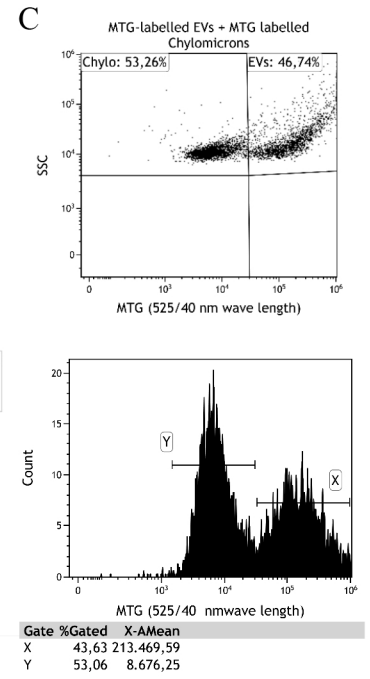

## Plasma EVs

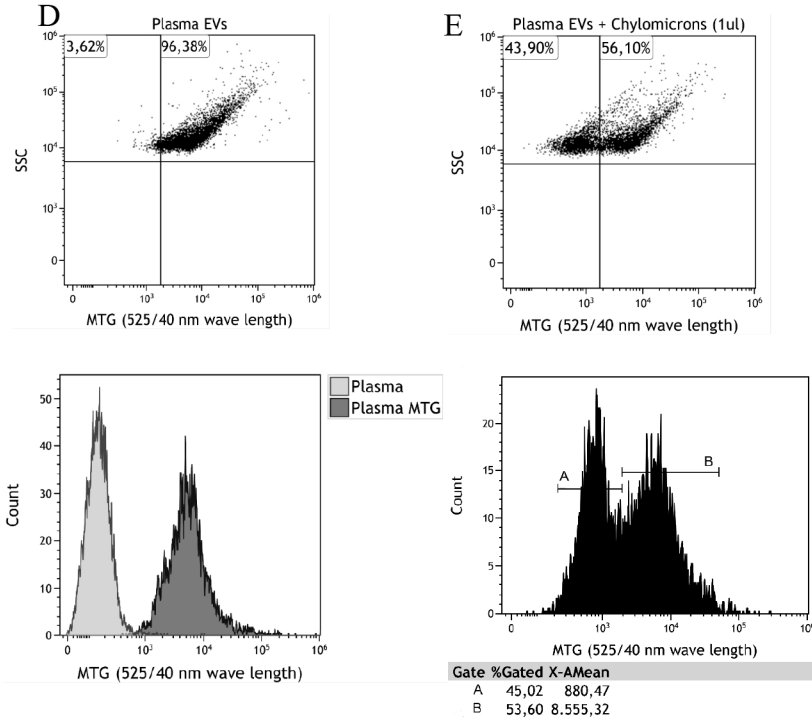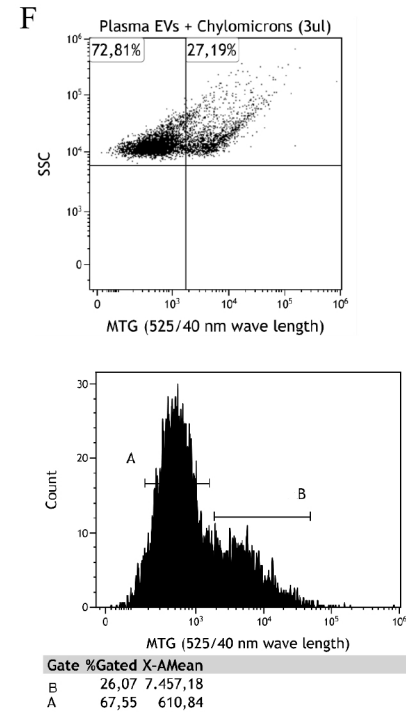

Supplement: Supplementary file 7 — Additional file 7. [file 12974_2024_3148_MOESM7_ESM.pdf]

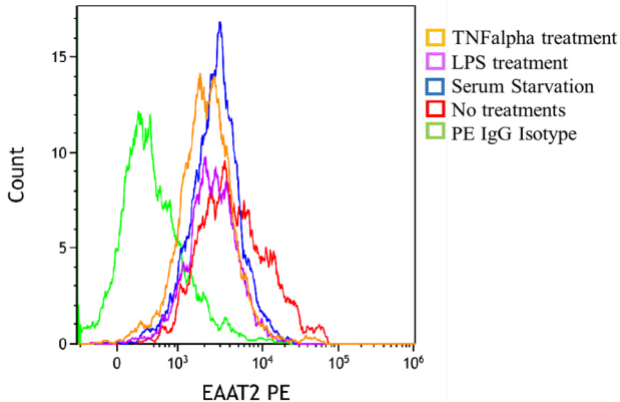

Supplement: Supplementary file 8 — Additional file 8. [file 12974_2024_3148_MOESM8_ESM.pdf]

Proteomic analysis of 20.000g and 100.000g plasma fractions from 3  
RRMS patients in Relapse

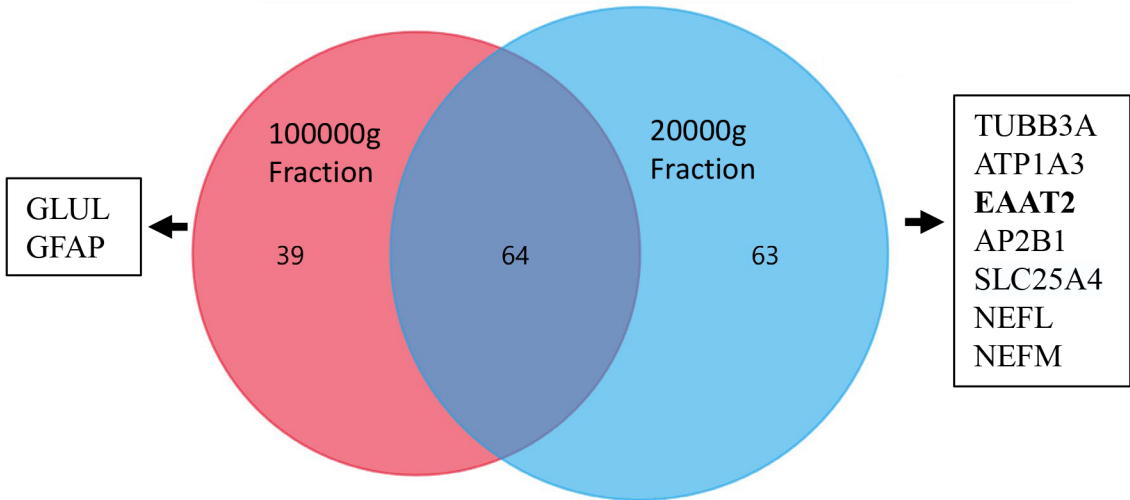

Supplement: Supplementary file 9 — Additional file 9. [file 12974_2024_3148_MOESM9_ESM.pdf]
