# Supplementary material for: Proteomic profile of extracellular vesicles from plasma and CSF of multiple sclerosis patients reveals disease activity-associated EAAT2
Source: J Neuroinflammation. 2024 Sep 2;21:217. doi: 10.1186/s12974-024-03148-x (PMC11370133; doi:10.1186/s12974-024-03148-x)
Supplement: Supplementary file 2 — Additional file 2. [file 12974_2024_3148_MOESM2_ESM.docx]

**Supplementary Material**

**Supplementary methods**

**SEC plasma EV purification**

Sephacryl S-500 High Resolution (GE Healthcare) column 26/60 (320 mL column volume), was used to isolate plasma EVs by Size exclusion chromatography^1^. 6 mL of individual PPP samples was loaded on the column and 0.22 µm-filtered PBS was used as running buffer, according to the manufacturer’s instructions. The flow rate was 1ml/min and the elution volume was collected in 6 fractions, each containing 40 ml. The 6 SEC fractions were sequentially centrifugated at 20.000g and then at 100.000g for two hours. The pellets of single SEC fractions obtained by each centrifugation step were washed and resuspended in PBS to analyse the EV presence by the determination of protein concentration using Bradford protein assay. The particle-size distribution and concentration of SEC fractions containing the major protein concentration were analysed by NanoSight.

**SEC CSF EV purification**

Sephacryl S-500 High Resolution (GE Healthcare) column 16/60 (120 mL volume column) was used to isolate CSF EVs. 4 mL of individual CSF samples was loaded on the column and 0.22 µm-filtered PBS was used as running buffer, according to the manufacturer’s instructions. The flow rate was 0,5ml/min. and the elution volume was collected in 6 fractions, each containing 15 ml, that were centrifugated at 1000.000g for two hours. Due to low levels of EVs in CSF, the pellets of fractions were pooled, washed and resuspended in PBS to measure total protein concentration and analyse the EV presence by TEM.

**Chylomicron isolation and analysis**

Postprandial blood samples were collected from healthy subjects two hours after meal. PPP was obtained according to the protocol reported in Material and methods. Chylomicrons were isolated from PPP according to Don’s protocol. ^2^ After isolation, protein concentration of chylomicron samples was determined by using Bradford protein assay. Moreover, chylomicron samples were visualized by TEM and their particle size distribution and concentration were evaluated by NTA, according to the protocols described in Material and methods.

**Western blot analysis to assess SEC-purified plasma EVs and chylomicron sample quality**

SEC-purified plasma EVs after 20.000g, SEC-purified plasma EVs after 100.000g centrifugation and purified chylomicrons were analysed for exosome and lipoprotein markers in western blotting. For each sample 20 µg of proteins, determined using the Bradford protein assay (Bio-Rad, USA), was loaded on polyacrylamide gels. Proteins were separated on 10% pre-casted acrylamide gels (Invitrogen, Carlsbad, CA) and transferred to PVDF membranes. The membranes were blocked (5% milk and 0.05% Tween-20) for 2 hrs and incubated overnight with ApoB-specific antibody (AbClonal A1330), TSG101-specific antibody (AbClonal A1692), CD81-specific antibody (SantaCruz sc-166029), Alix-specific antibody (Santa Cruz sc-271975, as primary antibody. After washing in PBS, the secondary HRP-conjugated anti-rabbit IgG (Sigma-Aldrich) was added. Chemiluminescent detection of proteins was performed using ECL Plus reagent (Amersham).

**MTG labelling of EVs**

To identify EV population at flow cytometry, SEC-purified plasma EVs or PPP containing EVs were labeled with MITO Tracker Green FM (MTG) (Molecular Probes-Invitrogen) at 100 nM in PBS for 15 min. at RT or PKH67 Green Fluorescent Cell Linker Kit for General Cell Membrane Labeling (Sigma-Aldrich), according to manufacturer’s instructions.

**MTG labelling of chylomicrons**

1µl of purified chylomicron samples, containing 1 µg of proteins, was stained with MTG at 100 nM in PBS for 15 min. at RT. Moreover, 1µl or 3 µl of purified chylomicrons were added to 2 µg SEC-purified plasma EVs and 3 µl of PPP (containing not purified EVs) before MTG labelling.

**EV treatment with thiol-blocking reagents before MTG labelling**

SEC-purified plasma EVs (from two different samples) were diluted in PBS and treated for 30 min at 37°C with different oxidant and alkylating agents: 1mM 6,6’-dinitro 3,3’-dithiobenzoic acid (DTNB), 1 mM N-ethylmaleimide (NEM),1 mM diamede (DIA),10 mM t-butyl-hydroperoxide (t-BHP) and 10 mM Hydrogen peroxide (HP). At the end of the treatment, EVs were centrifugated (100.000g) for 20 min, resuspended in 100 µl PBS and MTG labelled for 15 min before flow cytometry analysis. Moreover, SEC-purified plasma EVs, from three different samples, were treated with 10 mM HP for 30 min at 37°C and stained with MTG.

**Detergent Treatment of EVs**

SEC-purified EVs or PPP containing EVs, were untreated or treated for 15 min at RT with 0.075% Triton-X100 in PBS. After detergent incubation, MTG was added to the samples 15 min before cytofluorimetric analysis. Flow-Count Fluorospheres (Beckman Coulter, California,USA) were used to determine the exact event number in EV gate.

**EAAT2 detection on EVs isolated from U251 and K562 cell line supernatants**

The U251 and K562 cell lines were cultured according to ATCC guidelines. The culture medium was supplemented with 10% exosome-depleted FBS ≥90% (Gibco). U251- and K562-derived EVs, isolated from cell-free culture supernatants by centrifugation (100.000g for 20 min), were used for EAAT2 detection in western blot analysis. Cultured U251 cells were stimulated with TNF-α (10 ng/mL), LPS (50 µg/mL) or serum starved, for the last 4 hrs of culture. Cell-free supernatant from treated and untreated U251 cells was used to isolate EVs for EAAT2 detection in flow cytometry. PE-conjugated rabbit IgG isotype (Bioss, Boston- Massachusetts USA) was used as negative control in flow cytometry.

**Supplementary Results**

**SEC-purified plasma EV characterization**

The strategy to obtain plasma EV purification is reported in Supplementary Fig.1A. Due to size and density heterogeneity of extracellular vesicles, in order to preserve large EV membrane integrity, the SEC fractions were centrifuged firstly at 20.000g and then at 100.00g to have as much information as possible applying the proteomic approach. Both SEC fractions centrifuged at 20.000g and 100.000g with greater protein contents were analysed by Nanoparticle Tracking Analysis (NTA). According to the results obtained by Bradford and NTA analysis, we pooled pellets of 2, 3 and 4 SEC-EV fractions after 20.000g centrifugation (SEC-EVs 20K) and fraction 3, 4 and 5 after 100.000g centrifugation (SEC-EVs 100K) (Supplementary Fig. 1B, 1C and 1D). Furthermore, in order to assess the purity of purified EV samples, SEC-EVs 20K and SEC-EVs 100K were analysed for exosome markers (Alix, CD81 and TSG101) and lipoprotein marker (ApoB) in western blot assay (Supplementary Fig. 3C and 3D). The presence of exosome markers in both purified EV samples confirmed the goodness of the purification strategy, although lipoprotein contaminations was observed.

**Proteomic analysis of SEC-purified plasma and CSF EVs**

The proteomic analysis of SEC-purified plasma and CSF EVs identified a repertoire of 250 and 152 proteins, respectively (Supplementary table 1 and 5). The list of proteins obtained from each plasma and CSF sample group were reported in Supplementary Tables 2, 3, 4 and Supplementary Tables 6 and 7, respectively.

Insert Supplementary tables 1,2,3,4,5,6,7 here.

Insert Supplementary Fig 2 here.

Insert images of SDS-PAGE gels here.

**Efficiency of MTG binding to EV free-thiol groups**

To identify EV population by FACS analysis, the flow cytometer was adjusted to cover the EV size range between 0.5 and 1 µm, as shown in Supplementary Fig. 4A.

MTG labelling reagent, covalently binding to proteins by reacting with free thiol groups of cysteine residues, was used as a simple fluorescent probe to rapidly identify EVs suspended in plasma without the need to isolate them before flow cytometry analysis. In order to assess the efficiency of the MTG labelling technique for EVs, the lipophilic PKH67 Green dye or MTG-green dye were used to stain SEC-purified plasma EVs and not purified plasma EVs. MTG and PKH67 fluorescent dyes detected the same size-based population of EVs, as indicated in Supplementary Fig. 4B. Moreover, the Triton X-100 treatment of SEC-purified plasma EVs or not purified EVs (PPP containing EVs), labelled with MTG, showed that most of EV population disappeared for 89% and 78% respectively, indicating that the thiol-based fluorescence labelling method is appropriate for EV staining (Supplementary Fig. 4C and 4D). To demonstrate the specificity of MTG binding to free thiol groups in EVs, various thiol-blocking reagents were used to treat different samples of SEC-purified plasma EVs. All reagents used for EV treatments, alkylating reagents (NEM, DTNB and DIA) and oxidant reagents (t-BHP and HP), caused a mean fluorescence intensity (MFI) decrease of the EV-MTG**^+^** (data not shown). In particular, in three different samples, the oxidant reagent HP was reported to significantly decrease the MFI of EV-MTG**^+^** compared to untreated EVs (Supplementary Fig. 4E).

**Specificity of MTG binding to EV**

The presence of lipoproteins in plasma could interfere with the identification of EV population in the 0,5-1µm gate in flow cytometry, therefore MTG binding to chylomicrons was evaluated. Purified chylomicron characterization was performed by TEM, NTA and western blot analysis (Supplementary Fig. 3A, 3B and 3C). Although both purified chylomicrons and EVs were stained by MTG in the 0,5-1µm gate, SEC-purified EVs showed a higher mean intensity of fluorescence (MFI) than chylomicrons, as demonstrated in flow cytometry experiments where chylomicrons were added to SEC-purified EVs (Supplementary Fig. 5A, 5B and 5C). Moreover, the same result was obtained when difference concentrations of chylomicrons were added to plasma samples (Supplementary Fig. 5D, 5E and 5F).

Insert Supplementary Fig. 6 here

Insert Supplementary Fig. 7 here.

Insert Supplementary table 8 here.

**Supplementary References**

1. Smalley DM, Root KE, Cho H, et al. Proteomic discovery of 21 proteins expressed in human plasma-derived but not platelet-derived microparticles. Thromb Haemost. 2007; 97(1):67-80.
2. Don W, Wang X, Wu J, et al. The early effects of sleeve gastrectomy on postprandial chylomicron triglycerides during the progression of type 2 diabetes. Clin Chim Acta 2023; 549:117558. doi: 10.1016/j.cca.2023.117558.

**Supplementary Figure legends**

**Supplemantary Figure1. SEC-purification process of plasma EVs and SEC-EV fraction characterization.** (**A**) Schematic diagram of the EV purification process. (**B**) Protein concentration of SEC fractions after 20.000g and 100.000g centrifugation, determined by Bradford assay. Mean + SD of 3 independent samples of SEC-purified plasma EVs are shown. (**C**) Particle concentrations in SEC fractions by NTA analysis of 5 consecutive determinations of a representative sample of SEC-purified plasma EVs. (**D**) NTA analysis of SEC fractions after 20.000g and 100.000g centrifugation of a representative sample.

**Supplementary Figure 2**. **Pairwise comparison of shared EV proteins**. The matrix charts showing pairwise comparison of shared EV proteins (expressed both as a number and a percentage) between subjects belonging to the same group analysed. The chart was generated using the FunRich tool available at <http://www.funrich.org>.

A1-6, B1-5, H1-5, L1-4, M1-3: patient’s identification letter.

**Supplementary Figure 3. Characterization of plasma SEC-EVs 20K and SEC-EVs 100K** **and purified chylomicrons.** (**A**) Representative images of purified chylomicrons obtained by TEM. (**B**) NTA analysis of purified chylomicrons of a representative sample. (**C**) Representative Western blot of SEC-purified EVs and purified chylomicrons for ApoB detection. Lane K: purified chylomicrons; Lane EV: SEC-purified EVs; Lane M: Marker. (**D**) Representative Western blot of SEC-EVs 20K, SEC-EVs 100K and purified chylomicrons for Alix, CD81 and TSG101 detection. Lane1: purified chylomicrons; Lane 2: SEC-EVs 20K; Lane 3: SEC-EVs 100K; Lane M: marker.

**Supplementary Figure 4**. **Flow cytometric detection of plasma EVs**. (**A**) Gating strategy: on the left, forward scatter distribution histogram of a mixture of monodisperse beads of three different diameters ranging between 0.5 and 1 µm which were used as size reference beads to construct the EVs gate within the sensitivity limit of the cytometer. On the right, an illustrative scattergram of SEC-purified plasma EVs with size reference beads for EVs gate setting. Only events in the EVs size range between 0.5 and 1 um were included in the analysis. (**B**) Labelling of SEC-purified EVs and plasma EVs with PKH67 or MTG green dyes. Unlabelled SEC-purified EVs and plasma EVs were used as control. (**C**) (**D**) Effects of Triton X-100 treatment on SEC-purified or not purified (PPP) plasma EVs. On the left representative population of EVs in 0,5-1 µm gate (untreated EVs). In the middle representative population of EVs after 0,075% Triton X-100 treatment. On the right, overlay histogram of MTG-positive EV percentage before (grey) and after (black) Triton X-100 treatment. The event number of each EV sample was determined by employing Flow-Count fluorosferes (Beckman Coulter) used as internal reference counting beads. (**E**) Specificity of MTG binding to EV free thiol groups. The fluorescence histogram showing MFI of MTG-labelled SEC-purified plasma EVs untreated or treated with 10 mM HP, from three different samples. The MFI values (reported in the figure) were confirmed by a second experiment using the same three samples. Student’s t- test showed a statistically significant difference (*p*≤ 0,01) between the two groups (untreated and HP treated EVs).

**Supplementary Figure 5. MTG property to discern EV and chylomicron populations.** (**A**) Representative dot plot and histogram showing the MTG-labelled SEC-purified plasma EVs in the 0,5-1 µm gate. Unlabelled SEC-purified plasma EVs and MTG in PBS are used as control. (**B**) Representative dot plot and histogram showing the MTG-labelled chylomicrons in the 0,5-1 µm gate. Unlabelled chylomicrons and MTG in PBS are used as control. (**C**) Representative dot plot and histogram showing a suspension obtained adding 1 µl of MTG-labelled chylomicrons to 2 µl of MTG-labelled SEC-purified EVs. (**D**) Representative dot plot and histogram showing the MTG-labelled plasma EVs in the 0,5-1 µm gate. Unlabelled plasma EVs and MTG in PBS are used as control. (**E**) and (**F**) Representative dot plot and histogram showing 1 µl and 3 µl of MTG-labelled chylomicrons added to 3 µl of MTG-labelled plasma EVs, respectively.

MFI values are reported.

**Supplementary Figure 6**. (**A**) Representative histogram of one out of two experiments showing flow cytometry analysis of EAAT2+EVs in MTG-positive gate in culture supernatant from untreated or treated U251 cell line.

**Supplementary Figure 7. Venn diagram of plasma SEC EV 20K and SEC EV 100K proteins.** Venn diagram showing common and unique proteins of SEC EV 20K and SEC EV 100K samples from 3 individual relapsing RRMS patients, obtained by proteomic analysis. The boxes report some of proteins associated with relapse phase.
