# Supplementary material for: Proteomic profile of extracellular vesicles from plasma and CSF of multiple sclerosis patients reveals disease activity-associated EAAT2
Source: J Neuroinflammation. 2024 Sep 2;21:217. doi: 10.1186/s12974-024-03148-x (PMC11370133; doi:10.1186/s12974-024-03148-x)
Supplement: Supplementary file 4 — Additional file 4. [file 12974_2024_3148_MOESM4_ESM.pdf]

A Plasma EVs relapsing phase

	A1	A2	A3	A4	A5	A6
A1						
A2	66 (43,4%)					
A3	49 (35,0%)	43 (39,1%)				
A4	60 (40,8%)	49 (40,2%)	42 (42,0%)			
A5	74 (51,0%)	55 (43,0%)	44 (40,0%)	55 (47,0%)		
A6	44 (33,3%)	37 (35,9%)	34 (44,2%)	34 (35,8%)	37 (35,6%)	

B Plasma EVs remitting phase

	B1	B2	B3	B4	B5
B1					
B2	45 (36,6%)				
B3	46 (39,3%)	41 (50,0%)			
B4	35 (31,5%)	35 (49,3%)	38 (60,3%)		
B5	49 (41,2%)	39 (43,8%)	47 (61,8%)	37 (53,6%)	

C Plasma EVs Healthy subjects

	H1	H2	H3	H4	H5
H1					
H2	73 (57,0%)				
H3	90 (64,7%)	69 (58,0%)			
H4	94 (68,6%)	73 (62,4%)	86 (65,2%)		
H5	78 (59,1%)	62 (57,9%)	69 (53,9%)	73 (57,9%)	

D CSF EVs relapsing phase

	L1	L2	L3	L4
L1				
L2	34 (32,1%)			
L3	28 (26,7%)	37 (52,9%)		
L4	36 (31,6%)	38 (44,2%)	36 (44,4%)	

E CSF EVs remitting phase

	M1	M2	M3
M1			
M2	54 (53,5%)		
M3	51 (55,4%)	80 (76,9%)	
