# Supplementary material for: Proteomic profile of extracellular vesicles from plasma and CSF of multiple sclerosis patients reveals disease activity-associated EAAT2
Source: J Neuroinflammation. 2024 Sep 2;21:217. doi: 10.1186/s12974-024-03148-x (PMC11370133; doi:10.1186/s12974-024-03148-x)
Supplement: Supplementary file 11 — Additional file 11. [file 12974_2024_3148_MOESM11_ESM.docx]

**Supplementary table 2**. List of proteins identified by proteomic analysis of SEC-purified EVs

from plasma samples of 6 RRMS patients in relapse.

|  | Accession number | Gene Names | Protein Names |
| --- | --- | --- | --- |
| 1 | P31946 | YWHAB | 14-3-3 protein beta/alpha |
| 2 | P62258 | YWHAE | 14-3-3 protein epsilon |
| 3 | P61981 | YWHAG | 14-3-3 protein gamma |
| 4 | P27348 | YWHAQ | 14-3-3 protein theta |
| 5 | P63104 | YWHAZ | 14-3-3 protein zeta/delta |
| 6 | P09543 | CNP | 2',3'-cyclic-nucleotide 3'-phosphodiesterase |
| 7 | P80404 | ABAT, GABAT | 4-aminobutyrate aminotransferase, mitochondrial |
| 8 | P62736 | ACTA2, ACTSA, ACTVS, GIG46 | Actin, aortic smooth muscle |
| 9 | P60709 | ACTB | Actin, cytoplasmic 1 |
| 10 | P12235 | SLC25A4, AAC1, ANT1 | ADP/ATP translocase 1 |
| 11 | P12236 | SLC25A6, AAC3, ANT3, CDABP0051 | ADP/ATP translocase 3 |
| 12 | P43652 | AFM, ALB2, ALBA | Afamin |
| 13 | P02768 | ALB, GIG20, | Albumin |
| 14 | P02763 | ORM1, AGP1 | Alpha-1-acid glycoprotein 1 |
| 15 | P19652 | ORM2, AGP2 | Alpha-1-acid glycoprotein 2 |
| 16 | P01011 | SERPINA3, AACT, GIG24, GIG25 | Alpha-1-antichymotrypsin |
| 17 | P01009 | SERPINA1, AAT, PI, PRO0684, PRO2209 | Alpha-1-antitrypsin |
| 18 | P02765 | AHSG, FETUA, PRO2743 | Alpha-2-HS-glycoprotein |
| 19 | P01023 | A2M, CPAMD5, FWP007 | Alpha-2-macroglobulin |
| 20 | A8K2U0 | A2ML1, CPAMD9 | Alpha-2-macroglobulin-like protein 1 |
| 21 | P12814 | ACTN1 | Alpha-actinin-1 |
| 22 | P06733 | ENO1, ENO1L1, MBPB1, MPB1 | Alpha-enolase |
| 23 | Q16352 | INA, NEF5 | Alpha-internexin |
| 24 | P15144 | ANPEP, APN, CD13, PEPN | Aminopeptidase N |
| 25 | P01019 | AGT, SERPINA8 | Angiotensinogen |
| 26 | P07355 | ANXA2, ANX2, ANX2L4, CAL1H, LPC2D | Annexin A2 |
| 27 | P08758 | ANXA5, ANX5, ENX2, PP4 | Annexin A5 |
| 28 | P01008 | SERPINC1, AT3, PRO0309 | Antithrombin-III |
| 29 | O95782 | AP2A1, ADTAA, CLAPA1 | AP-2 complex subunit alpha-1 |
| 30 | P63010 | AP2B1, ADTB2, CLAPB1 | AP-2 complex subunit beta |
| 31 | P02647 | APOA1 | Apolipoprotein A-I |
| 32 | P06727 | APOA4 | Apolipoprotein A-IV |
| 33 | P04114 | APOB | Apolipoprotein B-100 |
| 34 | P02656 | APOC3 | Apolipoprotein C-III |
| 35 | P02649 | APOE | Apolipoprotein E |
| 36 | O14791 | APOL1, APOL | Apolipoprotein L1 |
| 37 | P08519 | LPA | Apolipoprotein(a) |
| 38 | Q9UKV3 | ACIN1, ACINUS, KIAA0670 | Apoptotic chromatin condensation inducer in the nucleus |
| 39 | P05089 | ARG1 | Arginase-1 |
| 40 | P25705 | ATP5F1A, ATP5A, ATP5A1, ATP5AL2, ATPM | ATP synthase subunit alpha, mitochondrial |
| 41 | P06576 | ATP5F1B, ATP5B, ATPMB, ATPSB | ATP synthase subunit beta, mitochondrial |
| 42 | P02730 | SLC4A1, AE1, DI, EPB3 | Band 3 anion transport protein |
| 43 | P02749 | APOH, B2G1 | Beta-2-glycoprotein 1 |
| 44 | Q562R1 | ACTBL2 | Beta-actin-like protein 2 |
| 45 | Q9HBI1 | PARVB, CGI-56 | Beta-parvin |
| 46 | P04003 | C4BPA, C4BP | C4b-binding protein alpha chain |
| 47 | P20851 | C4BPB | C4b-binding protein beta chain |
| 48 | Q9UQM7 | CAMK2A, CAMKA, KIAA0968 | Calcium/calmodulin-dependent protein kinase type II subunit alpha |
| 49 | Q13554 | CAMK2B, CAM2, CAMK2, CAMKB | Calcium/calmodulin-dependent protein kinase type II subunit beta |
| 50 | P22792 | CPN2, ACBP | Carboxypeptidase N subunit 2 |
| 51 | P31944 | CASP14 | Caspase-14 |
| 52 | P07858 | CTSB, CPSB | Cathepsin B |
| 53 | P07339 | CTSD, CPSD | Cathepsin D |
| 54 | O95810 | CAVIN2, SDPR | Caveolae-associated protein 2 |
| 55 | O43866 | CD5L, API6, UNQ203/PRO229 | CD5 antigen-like |
| 56 | P00450 | CP | Ceruloplasmin |
| 57 | Q00610 | CLTC, CLH17, CLTCL2, KIAA0034 | Clathrin heavy chain 1 |
| 58 | P10909 | CLU, APOJ, CLI, KUB1, AAG4 | Clusterin |
| 59 | P00488 | F13A1, F13A | Coagulation factor XIII A chain |
| 60 | P02745 | C1QA | Complement C1q subcomponent subunit A |
| 61 | P02746 | C1QB | Complement C1q subcomponent subunit B |
| 62 | P02747 | C1QC, C1QG | Complement C1q subcomponent subunit C |
| 63 | P00736 | C1R | Complement C1r subcomponent |
| 64 | P09871 | C1S | Complement C1s subcomponent |
| 65 | P01024 | C3, CPAMD1 | Complement C3 |
| 66 | P0C0L4 | C4A, CO4, CPAMD2 | Complement C4-A |
| 67 | P01031 | C5, CPAMD4 | Complement C5 |
| 68 | P13671 | C6 | Complement component C6 |
| 69 | P07358 | C8B | Complement component C8 beta chain |
| 70 | P02748 | C9 | Complement component C9 |
| 71 | P00751 | CFB, BF, BFD | Complement factor B |
| 72 | P08603 | CFH, HF, HF1, HF2 | Complement factor H |
| 73 | Q03591 | CFHR1, CFHL, CFHL1, CFHL1P, CFHR1P, FHR1, HFL1, HFL2 | Complement factor H-related protein 1 |
| 74 | P05156 | CFI, IF | Complement factor I |
| 75 | P02741 | CRP, PTX1 | C-reactive protein |
| 76 | P12277 | CKB, CKBB | Creatine kinase B-type |
| 77 | P81605 | DCD, AIDD, DSEP | Dermcidin |
| 78 | Q02413 | DSG1, CDHF4 | Desmoglein-1 |
| 79 | P15924 | DSP | Desmoplakin |
| 80 | Q16555 | DPYSL2, CRMP2, ULIP2 | Dihydropyrimidinase-related protein 2 |
| 81 | O14672 | ADAM10, KUZ, MADM | Disintegrin and metalloproteinase domain-containing protein 10 |
| 82 | O95147 | DUSP14, MKP6 | Dual specificity protein phosphatase 14 |
| 83 | Q05193 | DNM1, DNM | Dynamin-1 |
| 84 | P43004 | SLC1A2, EAAT2, GLT1 | Excitatory amino acid transporter 2 |
| 85 | P15311 | EZR, VIL2 | Ezrin |
| 86 | Q01469 | FABP5 | Fatty acid-binding protein 5 |
| 87 | Q86UX7 | FERMT3, KIND3, MIG2B, URP2 | Fermitin family homolog 3 |
| 88 | P02671 | FGA | Fibrinogen alpha chain |
| 89 | P02675 | FGB | Fibrinogen beta chain |
| 90 | P02679 | FGG, PRO2061 | Fibrinogen gamma chain |
| 91 | P02751 | FN1, FN | Fibronectin |
| 92 | O00602 | FCN1, FCNM | Ficolin-1 |
| 93 | Q15485 | FCN2, FCNL | Ficolin-2 |
| 94 | O75636 | FCN3, FCNH, HAKA1 | Ficolin-3 |
| 95 | Q5D862 | FLG2, IFPS | Filaggrin-2 |
| 96 | P21333 | FLNA, FLN, FLN1 | Filamin-A |
| 97 | Q08380 | LGALS3BP, M2BP | Galectin-3-binding protein |
| 98 | P06396 | GSN | Gelsolin |
| 99 | P14136 | GFAP | Glial fibrillary acidic protein |
| 100 | P15104 | GLUL, GLNS | Glutamine synthetase |
| 101 | P04406 | GAPDH, GAPD, CDABP0047, OK/SW-cl.12 | Glyceraldehyde-3-phosphate dehydrogenase |
| 102 | P09471 | GNAO1 | Guanine nucleotide-binding protein G(o) subunit alpha |
| 103 | P00738 | HP | Haptoglobin |
| 104 | P00739 | HPR | Haptoglobin-related protein |
| 105 | P07900 | HSP90AA1, HSP90A, HSPC1, HSPCA | Heat shock protein HSP 90-alpha |
| 106 | P69905 | HBA1, HBA2 | Hemoglobin subunit alpha |
| 107 | P68871 | HBB | Hemoglobin subunit beta |
| 108 | P02790 | HPX | Hemopexin |
| 109 | P19367 | HK1 | Hexokinase-1 |
| 110 | P04196 | HRG | Histidine-rich glycoprotein |
| 111 | P04908 | H2AC4, H2AFM, HIST1H2AB, H2AC8, H2AFA, HIST1H2AE | Histone H2A type 1-B/E |
| 112 | P33778 | H2BC3, H2BFF, HIST1H2BB | Histone H2B type 1-B |
| 113 | Q9Y6R7 | FCGBP | IgGFc-binding protein |
| 114 | P01876 | IGHA1 | Immunoglobulin heavy constant alpha 1 |
| 115 | P01857 | IGHG1 | Immunoglobulin heavy constant gamma 1 |
| 116 | P01859 | IGHG2 | Immunoglobulin heavy constant gamma 2 |
| 117 | P01860 | IGHG3 | Immunoglobulin heavy constant gamma 3 |
| 118 | P01861 | IGHG4 | Immunoglobulin heavy constant gamma 4 |
| 119 | P01871 | IGHM | Immunoglobulin heavy constant mu |
| 120 | P01762 | IGHV3-11 | Immunoglobulin heavy variable 3-11 |
| 121 | P01766 | IGHV3-13 | Immunoglobulin heavy variable 3-13 |
| 122 | P01764 | IGHV3-23 | Immunoglobulin heavy variable 3-23 |
| 123 | P01768 | IGHV3-30 | Immunoglobulin heavy variable 3-30 |
| 124 | P01763 | IGHV3-48 | Immunoglobulin heavy variable 3-48 |
| 125 | P01767 | IGHV3-53 | Immunoglobulin heavy variable 3-53 |
| 126 | P01780 | IGHV3-7 | Immunoglobulin heavy variable 3-7 |
| 127 | P01591 | JCHAIN, IGCJ, IGJ | Immunoglobulin J chain |
| 128 | P01834 | IGKC | Immunoglobulin kappa constant |
| 129 | P01597 | IGKV1-39 | Immunoglobulin kappa variable 1-39 |
| 130 | P01602 | IGKV1-5 | Immunoglobulin kappa variable 1-5 |
| 131 | P01593 | IGKV1D-33 | Immunoglobulin kappa variable 1D-33 |
| 132 | P01615 | IGKV2D-28 | Immunoglobulin kappa variable 2D-28 |
| 133 | P01614 | IGKV2D-40 | Immunoglobulin kappa variable 2D-40 |
| 134 | P04433 | IGKV3-11 | Immunoglobulin kappa variable 3-11 |
| 135 | P01624 | IGKV3-15 | Immunoglobulin kappa variable 3-15 |
| 136 | P01619 | IGKV3-20 | Immunoglobulin kappa variable 3-20 |
| 137 | P06312 | IGKV4-1 | Immunoglobulin kappa variable 4-1 |
| 138 | P0DOY2 | IGLC2 | Immunoglobulin lambda constant 2 |
| 139 | P0DOY3 | IGLC3 | Immunoglobulin lambda constant 3 |
| 140 | P80748 | IGLV3-21 | Immunoglobulin lambda variable 3-21 |
| 141 | B9A064 | IGLL5 | Immunoglobulin lambda-like polypeptide 5 |
| 142 | Q14643 | ITPR1, INSP3R1 | Inositol 1,4,5-trisphosphate receptor type 1 |
| 143 | P08514 | ITGA2B, GP2B, ITGAB | Integrin alpha-IIb |
| 144 | P05106 | ITGB3, GP3A | Integrin beta-3 |
| 145 | P19827 | ITIH1, IGHEP1 | Inter-alpha-trypsin inhibitor heavy chain H1 |
| 146 | P19823 | ITIH2, IGHEP2 | Inter-alpha-trypsin inhibitor heavy chain H2 |
| 147 | Q14624 | ITIH4, IHRP, ITIHL1, PK120, PRO1851 | Inter-alpha-trypsin inhibitor heavy chain H4 |
| 148 | P14923 | JUP, CTNNG, DP3 | Junction plakoglobin |
| 149 | P29622 | SERPINA4, KST, PI4 | Kallistatin |
| 150 | P01042 | KNG1, BDK, KNG | Kininogen-1 |
| 151 | P18428 | LBP | Lipopolysaccharide-binding protein |
| 152 | P61626 | LYZ, LZM | Lysozyme C |
| 153 | P60201 | PLP1, PLP | Myelin proteolipid protein |
| 154 | P35579 | MYH9 | Myosin-9 |
| 155 | P28331 | NDUFS1 | NADH-ubiquinone oxidoreductase 75 kDa subunit, mitochondrial |
| 156 | P07196 | NEFL, NF68, NFL | Neurofilament light polypeptide |
| 157 | P07197 | NEFM, NEF3, NFM | Neurofilament medium polypeptide |
| 158 | P03952 | KLKB1, KLK3 | Plasma kallikrein |
| 159 | P05155 | SERPING1, C1IN, C1NH | Plasma protease C1 inhibitor |
| 160 | P00747 | PLG | Plasminogen |
| 161 | P16284 | PECAM1 | Platelet endothelial cell adhesion molecule |
| 162 | P02776 | PF4, CXCL4, SCYB4 | Platelet factor 4 |
| 163 | P16671 | CD36, GP3B, GP4 | Platelet glycoprotein 4 |
| 164 | P13224 | GP1BB | Platelet glycoprotein Ib beta chain |
| 165 | P08567 | PLEK, P47 | Pleckstrin |
| 166 | P01833 | PIGR | Polymeric immunoglobulin receptor |
| 167 | P07737 | PFN1 | Profilin-1 |
| 168 | Q07954 | LRP1, A2MR, APR | Prolow-density lipoprotein receptor-related protein 1 |
| 169 | P15309 | ACP3, ACPP | Prostatic acid phosphatase |
| 170 | P02760 | AMBP, HCP, ITIL | Protein AMBP |
| 171 | P31151 | S100A7, PSOR1, S100A7C | Protein S100-A7 |
| 172 | P06702 | S100A9, CAGB, CFAG, MRP14 | Protein S100-A9 |
| 173 | P22735 | TGM1, KTG | Protein-glutamine gamma-glutamyltransferase K |
| 174 | P00734 | F2 | Prothrombin |
| 175 | Q9BYX7 | POTEKP, ACTBL3, FKSG30 | Putative beta-actin-like protein 3 |
| 176 | P14618 | PKM, OIP3, PK2, PK3, PKM2 | Pyruvate kinase PKM |
| 177 | Q15404 | RSU1, RSP1 | Ras suppressor protein 1 |
| 178 | P62834 | RAP1A, KREV1 | Ras-related protein Rap-1A |
| 179 | P02787 | TF, PRO1400 | Serotransferrin |
| 180 | P02743 | APCS, PTX2 | Serum amyloid P-component |
| 181 | P05023 | ATP1A1 | Sodium/potassium-transporting ATPase subunit alpha-1 |
| 182 | P13637 | ATP1A3 | Sodium/potassium-transporting ATPase subunit alpha-3 |
| 183 | Q13813 | SPTAN1, NEAS, SPTA2 | Spectrin alpha chain, non-erythrocytic 1 |
| 184 | Q01082 | SPTBN1, SPTB2 | Spectrin beta chain, non-erythrocytic 1 |
| 185 | P27105 | STOM, BND7, EPB72 | Stomatin |
| 186 | P60880 | SNAP25, SNAP | Synaptosomal-associated protein 25 |
| 187 | P21579 | SYT1, SVP65, SYT | Synaptotagmin-1 |
| 188 | P61764 | STXBP1, UNC18A | Syntaxin-binding protein 1 |
| 189 | Q15833 | STXBP2, UNC18B | Syntaxin-binding protein 2 |
| 190 | Q9Y490 | TLN1, KIAA1027, TLN | Talin-1 |
| 191 | Q5TAX3 | TUT4, KIAA0191, ZCCHC11 | Terminal uridylyltransferase 4 |
| 192 | P02786 | TFRC | Transferrin receptor protein 1 |
| 193 | P55072 | VCP, HEL-220, HEL-S-70 | Transitional endoplasmic reticulum ATPase |
| 194 | P02766 | TTR, PALB | Transthyretin |
| 195 | Q71U36 | TUBA1A, TUBA3 | Tubulin alpha-1A chain |
| 196 | P68363 | TUBA1B | Tubulin alpha-1B chain |
| 197 | P07437 | TUBB, TUBB5, OK/SW-cl.56 | Tubulin beta chain |
| 198 | Q9H4B7 | TUBB1 | Tubulin beta-1 chain |
| 199 | Q13885 | TUBB2A, TUBB2 | Tubulin beta-2A chain |
| 200 | Q13509 | TUBB3, TUBB4 | Tubulin beta-3 chain |
| 201 | P04350 | TUBB4A, TUBB4, TUBB5 | Tubulin beta-4A chain |
| 202 | Q9BUF5 | TUBB6 | Tubulin beta-6 chain |
| 203 | P63027 | VAMP2, SYB2 | Vesicle-associated membrane protein 2 |
| 204 | P46459 | NSF | Vesicle-fusing ATPase |
| 205 | P18206 | VCL | Vinculin |
| 206 | P02774 | GC | Vitamin D-binding protein |
| 207 | P07225 | PROS1, PROS | Vitamin K-dependent protein S |
| 208 | P04004 | VTN | Vitronectin |
| 209 | P04275 | VWF, F8VWF | von Willebrand factor |
| 210 | Q93050 | ATP6V0A1, ATP6N1, ATP6N1A, VPP1 | V-type proton ATPase 116 kDa subunit a 1 |
