# Supplementary material for: Proteomic profile of extracellular vesicles from plasma and CSF of multiple sclerosis patients reveals disease activity-associated EAAT2
Source: J Neuroinflammation. 2024 Sep 2;21:217. doi: 10.1186/s12974-024-03148-x (PMC11370133; doi:10.1186/s12974-024-03148-x)
Supplement: Supplementary file 12 — Additional file 12. [file 12974_2024_3148_MOESM12_ESM.docx]

**Supplementary table 3.** List of proteins identified by proteomic analysis of SEC-purified EVs from plasma samples of 5 RRMS patients in remission.

|  | Accession number | Gene Names | Protein Names |
| --- | --- | --- | --- |
| 1 | P31946 | YWHAB | 14-3-3 protein beta/alpha |
| 2 | P62258 | YWHAE | 14-3-3 protein epsilon |
| 3 | Q04917 | YWHAH, YWHA1 | 14-3-3 protein eta |
| 4 | P61981 | YWHAG | 14-3-3 protein gamma |
| 5 | P27348 | YWHAQ | 14-3-3 protein theta |
| 6 | P63104 | YWHAZ | 14-3-3 protein zeta/delta |
| 7 | P62736 | ACTA2, ACTSA, ACTVS, GIG46 | Actin, aortic smooth muscle |
| 8 | P60709 | ACTB | Actin, cytoplasmic 1 |
| 9 | P84077 | ARF1 | ADP-ribosylation factor 1 |
| 10 | P02768 | ALB, GIG20, | Albumin |
| 11 | P02763 | ORM1, AGP1 | Alpha-1-acid glycoprotein 1 |
| 12 | P19652 | ORM2, AGP2 | Alpha-1-acid glycoprotein 2 |
| 13 | P01011 | SERPINA3, AACT, GIG24, GIG25 | Alpha-1-antichymotrypsin |
| 14 | P01009 | SERPINA1, AAT, PI, PRO0684, PRO2209 | Alpha-1-antitrypsin |
| 15 | P04217 | A1BG | Alpha-1B-glycoprotein |
| 16 | P01023 | A2M, CPAMD5, FWP007 | Alpha-2-macroglobulin |
| 17 | P12814 | ACTN1 | Alpha-actinin-1 |
| 18 | P06733 | ENO1, ENO1L1, MBPB1, MPB1 | Alpha-enolase |
| 19 | P01019 | AGT, SERPINA8 | Angiotensinogen |
| 20 | P01008 | SERPINC1, AT3, PRO0309 | Antithrombin-III |
| 21 | P02647 | APOA1 | Apolipoprotein A-I |
| 22 | P04114 | APOB | Apolipoprotein B-100 |
| 23 | P02649 | APOE | Apolipoprotein E |
| 24 | O14791 | APOL1, APOL | Apolipoprotein L1 |
| 25 | P08519 | LPA | Apolipoprotein(a) |
| 26 | P05089 | ARG1 | Arginase-1 |
| 27 | P02730 | SLC4A1, AE1, DI, EPB3 | Band 3 anion transport protein |
| 28 | P02749 | APOH, B2G1 | Beta-2-glycoprotein 1 |
| 29 | Q9HBI1 | PARVB, CGI-56 | Beta-parvin |
| 30 | P04003 | C4BPA, C4BP | C4b-binding protein alpha chain |
| 31 | P20851 | C4BPB | C4b-binding protein beta chain |
| 32 | P31944 | CASP14 | Caspase-14 |
| 33 | O95810 | CAVIN2, SDPR | Caveolae-associated protein 2 |
| 34 | O43866 | CD5L, API6, UNQ203/PRO229 | CD5 antigen-like |
| 35 | P00450 | CP | Ceruloplasmin |
| 36 | Q00610 | CLTC, CLH17, CLTCL2, KIAA0034 | Clathrin heavy chain 1 |
| 37 | P10909 | CLU, APOJ, CLI, KUB1, AAG4 | Clusterin |
| 38 | P00488 | F13A1, F13A | Coagulation factor XIII A chain |
| 39 | P23528 | CFL1, CFL | Cofilin-1 |
| 40 | P02745 | C1QA | Complement C1q subcomponent subunit A |
| 41 | P02746 | C1QB | Complement C1q subcomponent subunit B |
| 42 | P00736 | C1R | Complement C1r subcomponent |
| 43 | P09871 | C1S | Complement C1s subcomponent |
| 44 | P01024 | C3, CPAMD1 | Complement C3 |
| 45 | P0C0L4 | C4A, CO4, CPAMD2 | Complement C4-A |
| 46 | P13671 | C6 | Complement component C6 |
| 47 | P07360 | C8G | Complement component C8 gamma chain |
| 48 | P08603 | CFH, HF, HF1, HF2 | Complement factor H |
| 49 | P01040 | CSTA, STF1, STFA | Cystatin-A |
| 50 | P81605 | DCD, AIDD, DSEP | Dermcidin |
| 51 | Q02413 | DSG1, CDHF4 | Desmoglein-1 |
| 52 | P15924 | DSP | Desmoplakin |
| 53 | O14672 | ADAM10, KUZ, MADM | Disintegrin and metalloproteinase domain-containing protein 10 |
| 54 | Q86UX7 | FERMT3, KIND3, MIG2B, URP2 | Fermitin family homolog 3 |
| 55 | P02671 | FGA | Fibrinogen alpha chain |
| 56 | P02675 | FGB | Fibrinogen beta chain |
| 57 | P02679 | FGG, PRO2061 | Fibrinogen gamma chain |
| 58 | P02751 | FN1, FN | Fibronectin |
| 59 | Q15485 | FCN2, FCNL | Ficolin-2 |
| 60 | P21333 | FLNA, FLN, FLN1 | Filamin-A |
| 61 | P04075 | ALDOA, ALDA | Fructose-bisphosphate aldolase A |
| 62 | Q08380 | LGALS3BP, M2BP | Galectin-3-binding protein |
| 63 | P06396 | GSN | Gelsolin |
| 64 | P09211 | GSTP1, FAEES3, GST3 | Glutathione S-transferase P |
| 65 | P04406 | GAPDH, GAPD, CDABP0047, OK/SW-cl.12 | Glyceraldehyde-3-phosphate dehydrogenase |
| 66 | P04899 | GNAI2, GNAI2B | Guanine nucleotide-binding protein G(i) subunit alpha-2 |
| 67 | P50148 | GNAQ, GAQ | Guanine nucleotide-binding protein G(q) subunit alpha |
| 68 | P00738 | HP | Haptoglobin |
| 69 | P00739 | HPR | Haptoglobin-related protein |
| 70 | P07900 | HSP90AA1, HSP90A, HSPC1, HSPCA | Heat shock protein HSP 90-alpha |
| 71 | P69905 | HBA1, HBA2 | Hemoglobin subunit alpha |
| 72 | P68871 | HBB | Hemoglobin subunit beta |
| 73 | P02790 | HPX | Hemopexin |
| 74 | P04196 | HRG | Histidine-rich glycoprotein |
| 75 | P04439 | HLA-A, HLAA | HLA class I histocompatibility antigen, A alpha chain |
| 76 | Q9Y6R7 | FCGBP | IgGFc-binding protein |
| 77 | P01876 | IGHA1 | Immunoglobulin heavy constant alpha 1 |
| 78 | P01857 | IGHG1 | Immunoglobulin heavy constant gamma 1 |
| 79 | P01859 | IGHG2 | Immunoglobulin heavy constant gamma 2 |
| 80 | P01860 | IGHG3 | Immunoglobulin heavy constant gamma 3 |
| 81 | P01861 | IGHG4 | Immunoglobulin heavy constant gamma 4 |
| 82 | P01871 | IGHM | Immunoglobulin heavy constant mu |
| 83 | P01766 | IGHV3-13 | Immunoglobulin heavy variable 3-13 |
| 84 | P01764 | IGHV3-23 | Immunoglobulin heavy variable 3-23 |
| 85 | P01768 | IGHV3-30 | Immunoglobulin heavy variable 3-30 |
| 86 | P01767 | IGHV3-53 | Immunoglobulin heavy variable 3-53 |
| 87 | P01780 | IGHV3-7 | Immunoglobulin heavy variable 3-7 |
| 88 | P01591 | JCHAIN, IGCJ, IGJ | Immunoglobulin J chain |
| 89 | P01834 | IGKC | Immunoglobulin kappa constant |
| 90 | P01597 | IGKV1-39 | Immunoglobulin kappa variable 1-39 |
| 91 | P01602 | IGKV1-5 | Immunoglobulin kappa variable 1-5 |
| 92 | P01593 | IGKV1D-33 | Immunoglobulin kappa variable 1D-33 |
| 93 | P06310 | IGKV2-30 | Immunoglobulin kappa variable 2-30 |
| 94 | P01615 | IGKV2D-28 | Immunoglobulin kappa variable 2D-28 |
| 95 | P04433 | IGKV3-11 | Immunoglobulin kappa variable 3-11 |
| 96 | P01624 | IGKV3-15 | Immunoglobulin kappa variable 3-15 |
| 97 | P01619 | IGKV3-20 | Immunoglobulin kappa variable 3-20 |
| 98 | P06312 | IGKV4-1 | Immunoglobulin kappa variable 4-1 |
| 99 | P0DOY2 | IGLC2 | Immunoglobulin lambda constant 2 |
| 100 | P0DOY3 | IGLC3 | Immunoglobulin lambda constant 3 |
| 101 | P80748 | IGLV3-21 | Immunoglobulin lambda variable 3-21 |
| 102 | B9A064 | IGLL5 | Immunoglobulin lambda-like polypeptide 5 |
| 103 | P17301 | ITGA2, CD49B | Integrin alpha-2 |
| 104 | P23229 | ITGA6 | Integrin alpha-6 |
| 105 | P08514 | ITGA2B, GP2B, ITGAB | Integrin alpha-IIb |
| 106 | P05556 | ITGB1, FNRB, MDF2, MSK12 | Integrin beta-1 |
| 107 | P05106 | ITGB3, GP3A | Integrin beta-3 |
| 108 | P19827 | ITIH1, IGHEP1 | Inter-alpha-trypsin inhibitor heavy chain H1 |
| 109 | Q14624 | ITIH4, IHRP, ITIHL1, PK120, PRO1851 | Inter-alpha-trypsin inhibitor heavy chain H4 |
| 110 | P14923 | JUP, CTNNG, DP3 | Junction plakoglobin |
| 111 | P01042 | KNG1, BDK, KNG | Kininogen-1 |
| 112 | P02788 | LTF, GIG12, LF | Lactotransferrin |
| 113 | P18428 | LBP | Lipopolysaccharide-binding protein |
| 114 | P61626 | LYZ, LZM | Lysozyme C |
| 115 | O75556 | SCGB2A1, LIPHC, MGB2, UGB3 | Mammaglobin-B |
| 116 | Q13201 | MMRN1, ECM, EMILIN4, GPIA*, MMRN | Multimerin-1 |
| 117 | P19105 | MYL12A, MLCB, MRLC3, RLC | Myosin regulatory light chain 12A |
| 118 | P35580 | MYH10 | Myosin-10 |
| 119 | P35579 | MYH9 | Myosin-9 |
| 120 | O15527 | OGG1, MMH, MUTM, OGH1 | N-glycosylase/DNA lyase |
| 121 | P62937 | PPIA, CYPA | Peptidyl-prolyl cis-trans isomerase A |
| 122 | P03952 | KLKB1, KLK3 | Plasma kallikrein |
| 123 | P05155 | SERPING1, C1IN, C1NH | Plasma protease C1 inhibitor |
| 124 | P16284 | PECAM1 | Platelet endothelial cell adhesion molecule |
| 125 | P02776 | PF4, CXCL4, SCYB4 | Platelet factor 4 |
| 126 | P16671 | CD36, GP3B, GP4 | Platelet glycoprotein 4 |
| 127 | P07359 | GP1BA | Platelet glycoprotein Ib alpha chain |
| 128 | P13224 | GP1BB | Platelet glycoprotein Ib beta chain |
| 129 | P08567 | PLEK, P47 | Pleckstrin |
| 130 | P01833 | PIGR | Polymeric immunoglobulin receptor |
| 131 | P07737 | PFN1 | Profilin-1 |
| 132 | Q07954 | LRP1, A2MR, APR | Prolow-density lipoprotein receptor-related protein 1 |
| 133 | P31151 | S100A7, PSOR1, S100A7C | Protein S100-A7 |
| 134 | P00734 | F2 | Prothrombin |
| 135 | Q9BYX7 | POTEKP, ACTBL3, FKSG30 | Putative beta-actin-like protein 3 |
| 136 | P14618 | PKM, OIP3, PK2, PK3, PKM2 | Pyruvate kinase PKM |
| 137 | P31150 | GDI1, GDIL, OPHN2, RABGDIA, XAP4 | Rab GDP dissociation inhibitor alpha |
| 138 | Q15404 | RSU1, RSP1 | Ras suppressor protein 1 |
| 139 | P61026 | RAB10 | Ras-related protein Rab-10 |
| 140 | P11234 | RALB | Ras-related protein Ral-B |
| 141 | P62834 | RAP1A, KREV1 | Ras-related protein Rap-1A |
| 142 | P61224 | RAP1B, OK/SW-cl.11 | Ras-related protein Rap-1b |
| 143 | Q12913 | PTPRJ, DEP1 | Receptor-type tyrosine-protein phosphatase eta |
| 144 | P02787 | TF, PRO1400 | Serotransferrin |
| 145 | P02743 | APCS, PTX2 | Serum amyloid P-component |
| 146 | P05023 | ATP1A1 | Sodium/potassium-transporting ATPase subunit alpha-1 |
| 147 | P27105 | STOM, BND7, EPB72 | Stomatin |
| 148 | Q15833 | STXBP2, UNC18B | Syntaxin-binding protein 2 |
| 149 | Q9Y490 | TLN1, KIAA1027, TLN | Talin-1 |
| 150 | P07996 | THBS1, TSP, TSP1 | Thrombospondin-1 |
| 151 | P02786 | TFRC | Transferrin receptor protein 1 |
| 152 | P37802 | TAGLN2, KIAA0120, CDABP0035 | Transgelin-2 |
| 153 | P55072 | VCP, HEL-220, HEL-S-70 | Transitional endoplasmic reticulum ATPase |
| 154 | Q71U36 | TUBA1A, TUBA3 | Tubulin alpha-1A chain |
| 155 | P68363 | TUBA1B | Tubulin alpha-1B chain |
| 156 | Q9H4B7 | TUBB1 | Tubulin beta-1 chain |
| 157 | Q13885 | TUBB2A, TUBB2 | Tubulin beta-2A chain |
| 158 | P04350 | TUBB4A, TUBB4, TUBB5 | Tubulin beta-4A chain |
| 159 | P18206 | VCL | Vinculin |
| 160 | P02774 | GC | Vitamin D-binding protein |
| 161 | P07225 | PROS1, PROS | Vitamin K-dependent protein S |
| 162 | P04004 | VTN | Vitronectin |
| 163 | P04275 | VWF, F8VWF | von Willebrand factor |
