# Supplementary material for: Proteomic profile of extracellular vesicles from plasma and CSF of multiple sclerosis patients reveals disease activity-associated EAAT2
Source: J Neuroinflammation. 2024 Sep 2;21:217. doi: 10.1186/s12974-024-03148-x (PMC11370133; doi:10.1186/s12974-024-03148-x)
Supplement: Supplementary file 15 — Additional file 15. [file 12974_2024_3148_MOESM15_ESM.docx]

**Supplementary table** **6**. List of proteins obtained by proteomic analysis of SEC-purified EVs from CSF samples of 4 RRMS patients in relapse.

|  | Accession number | Gene Names | Protein Names |
| --- | --- | --- | --- |
| 1 | P62258 | YWHAE | 14-3-3 protein epsilon |
| 2 | P68032 | ACTC1, ACTC | Actin, alpha cardiac muscle 1 |
| 3 | P68133 | ACTA1, ACTA | Actin, alpha skeletal muscle |
| 4 | P60709 | ACTB | Actin, cytoplasmic 1 |
| 5 | P12235 | SLC25A4, AAC1, ANT1 | ADP/ATP translocase 1 |
| 6 | P05141 | SLC25A5, AAC2, ANT2 | ADP/ATP translocase 2 |
| 7 | P61204 | ARF3 | ADP-ribosylation factor 3 |
| 8 | P02768 | ALB, GIG20 | Albumin |
| 9 | P02763 | ORM1, AGP1 | Alpha-1-acid glycoprotein 1 |
| 10 | P01011 | SERPINA3, AACT, GIG24, GIG25 | Alpha-1-antichymotrypsin |
| 11 | P01009 | SERPINA1, AAT, PI, PRO0684, PRO2209 | Alpha-1-antitrypsin |
| 12 | P08697 | SERPINF2, AAP, PLI | Alpha-2-antiplasmin |
| 13 | P02765 | AHSG, FETUA, PRO2743 | Alpha-2-HS-glycoprotein |
| 14 | P01023 | A2M, CPAMD5, FWP007 | Alpha-2-macroglobulin |
| 15 | P35609 | ACTN2 | Alpha-actinin-2 |
| 16 | P51693 | APLP1 | Amyloid beta precursor like protein 1 |
| 17 | P01019 | AGT, SERPINA8 | Angiotensinogen |
| 18 | P01008 | SERPINC1, AT3, PRO0309 | Antithrombin-III |
| 19 | O94973 | AP2A2, ADTAB, CLAPA2, HIP9, HYPJ, KIAA0899 | AP-2 complex subunit alpha-2 |
| 20 | P63010 | AP2B1, ADTB2, CLAPB1 | AP-2 complex subunit beta |
| 21 | P02647 | APOA1 | Apolipoprotein A-I |
| 22 | P06727 | APOA4 | Apolipoprotein A-IV |
| 23 | P04114 | APOB | Apolipoprotein B-100 |
| 24 | P02649 | APOE | Apolipoprotein E |
| 25 | P25705 | ATP5F1A, ATP5A, ATP5A1, ATP5AL2, ATPM | ATP synthase subunit alpha, mitochondrial |
| 26 | P06576 | ATP5F1B, ATP5B, ATPMB, ATPSB | ATP synthase subunit beta, mitochondrial |
| 27 | P02730 | SLC4A1, AE1, DI, EPB3 | Band 3 anion transport protein |
| 28 | O43505 | B4GAT1, B3GNT1, B3GNT6 | Beta-1,4-glucuronyltransferase 1 |
| 29 | P02749 | APOH, B2G1 | Beta-2-glycoprotein 1 |
| 30 | Q96KN2 | CNDP1, CN1, CPGL2, UNQ1915/PRO4380 | Beta-Ala-His dipeptidase |
| 31 | P04003 | C4BPA, C4BP | C4b-binding protein alpha chain |
| 32 | Q9UQM7 | CAMK2A, CAMKA, KIAA0968 | Calcium/calmodulin-dependent protein kinase type II subunit alpha |
| 33 | P31944 | CASP14 | Caspase-14 |
| 34 | O43866 | CD5L, API6, UNQ203/PRO229 | CD5 antigen-like |
| 35 | P00450 | CP | Ceruloplasmin |
| 36 | Q00610 | CLTC, CLH17, CLTCL2, KIAA0034 | Clathrin heavy chain 1 |
| 37 | P10909 | CLU, APOJ, CLI, KUB1, AAG4 | Clusterin |
| 38 | P02745 | C1QA | Complement C1q subcomponent subunit A |
| 39 | P02746 | C1QB | Complement C1q subcomponent subunit B |
| 40 | P09871 | C1S | Complement C1s subcomponent |
| 41 | P01024 | C3, CPAMD1 | Complement C3 |
| 42 | P0C0L4 | C4A, CO4, CPAMD2 | Complement C4-A |
| 43 | P0C0L5 | C4B, CO4, CPAMD3, C4B_2 | Complement C4-B |
| 44 | P01031 | C5, CPAMD4 | Complement C5 |
| 45 | P10643 | C7 | Complement component C7 |
| 46 | P00751 | CFB, BF, BFD | Complement factor B |
| 47 | P08603 | CFH, HF, HF1, HF2 | Complement factor H |
| 48 | P05156 | CFI, IF | Complement factor I |
| 49 | Q12860 | CNTN1 | Contactin-1 |
| 50 | P12277 | CKB, CKBB | Creatine kinase B-type |
| 51 | P01034 | CST3 | Cystatin-C |
| 52 | Q02413 | DSG1, CDHF4 | Desmoglein-1 |
| 53 | P15924 | DSP | Desmoplakin |
| 54 | Q13822 | ENPP2, ATX, PDNP2 | Ectonucleotide pyrophosphatase/phosphodiesterase family member 2 |
| 55 | Q12805 | EFEMP1, FBLN3, FBNL | EGF-containing fibulin-like extracellular matrix protein 1 |
| 56 | O75746 | SLC25A12, AGC1, ARALAR1 | Electrogenic aspartate/glutamate antiporter SLC25A12, mitochondrial |
| 57 | P43004 | SLC1A2, EAAT2, GLT1 | Excitatory amino acid transporter 2 |
| 58 | P02671 | FGA | Fibrinogen alpha chain |
| 59 | P02675 | FGB | Fibrinogen beta chain |
| 60 | P02679 | FGG, PRO2061 | Fibrinogen gamma chain |
| 61 | P02751 | FN1, FN | Fibronectin |
| 62 | P23142 | FBLN1, PP213 | Fibulin-1 |
| 63 | Q15485 | FCN2, FCNL | Ficolin-2 |
| 64 | Q5D862 | FLG2, IFPS | Filaggrin-2 |
| 65 | Q08380 | LGALS3BP, M2BP | Galectin-3-binding protein |
| 66 | P06396 | GSN | Gelsolin |
| 67 | P14136 | GFAP | Glial fibrillary acidic protein |
| 68 | P04406 | GAPDH, GAPD, CDABP0047, OK/SW-cl.12 | Glyceraldehyde-3-phosphate dehydrogenase |
| 69 | P09471 | GNAO1 | Guanine nucleotide-binding protein G(o) subunit alpha |
| 70 | P00738 | HP | Haptoglobin |
| 71 | P08238 | HSP90AB1, HSP90B, HSPC2, HSPCB | Heat shock protein HSP 90-beta |
| 72 | P68871 | HBB | Hemoglobin subunit beta |
| 73 | P02790 | HPX | Hemopexin |
| 74 | P05546 | SERPIND1, HCF2 | Heparin cofactor 2 |
| 75 | P19367 | HK1 | Hexokinase-1 |
| 76 | P04196 | HRG | Histidine-rich glycoprotein |
| 77 | Q9Y6R7 | FCGBP | IgGFc-binding protein |
| 78 | P01876 | IGHA1 | Immunoglobulin heavy constant alpha 1 |
| 79 | P01857 | IGHG1 | Immunoglobulin heavy constant gamma 1 |
| 80 | P01859 | IGHG2 | Immunoglobulin heavy constant gamma 2 |
| 81 | P01860 | IGHG3 | Immunoglobulin heavy constant gamma 3 |
| 82 | P01861 | IGHG4 | Immunoglobulin heavy constant gamma 4 |
| 83 | P01871 | IGHM | Immunoglobulin heavy constant mu |
| 84 | P01834 | IGKC | Immunoglobulin kappa constant |
| 85 | P01619 | IGKV3-20 | Immunoglobulin kappa variable 3-20 |
| 86 | P06312 | IGKV4-1 | Immunoglobulin kappa variable 4-1 |
| 87 | P0CG04 | IGLC1 | Immunoglobulin lambda constant 1 |
| 88 | P0DOY2 | IGLC2 | Immunoglobulin lambda constant 2 |
| 89 | Q14624 | ITIH4, IHRP, ITIHL1, PK120, PRO1851 | Inter-alpha-trypsin inhibitor heavy chain H4 |
| 90 | P14923 | JUP, CTNNG, DP3 | Junction plakoglobin |
| 91 | Q14764 | MVP, LRP | Major vault protein |
| 92 | Q02978 | SLC25A11, SLC20A4 | Mitochondrial 2-oxoglutarate/malate carrier protein |
| 93 | P08571 | CD14 | Monocyte differentiation antigen CD14 |
| 94 | P02686 | MBP | Myelin basic protein |
| 95 | P12882 | MYH1 | Myosin-1 |
| 96 | Q9UKX2 | MYH2, MYHSA2 | Myosin-2 |
| 97 | P11055 | MYH3 | Myosin-3 |
| 98 | P12883 | MYH7, MYHCB | Myosin-7 |
| 99 | P13535 | MYH8 | Myosin-8 |
| 100 | P35579 | MYH9 | Myosin-9 |
| 101 | Q92823 | NRCAM, KIAA0343 | Neuronal cell adhesion molecule |
| 102 | P36955 | SERPINF1, PEDF, PIG35 | Pigment epithelium-derived factor |
| 103 | P05155 | SERPING1, C1IN, C1NH | Plasma protease C1 inhibitor |
| 104 | P00747 | PLG | Plasminogen |
| 105 | P08567 | PLEK, P47 | Pleckstrin |
| 106 | P12273 | PIP, GCDFP15, GPIP4 | Prolactin-inducible protein |
| 107 | P41222 | PTGDS, PDS | Prostaglandin-H2 D-isomerase |
| 108 | P31151 | S100A7, PSOR1, S100A7C | Protein S100-A7 |
| 109 | P14618 | PKM, OIP3, PK2, PK3, PKM2 | Pyruvate kinase PKM |
| 110 | P30153 | PPP2R1A | Serine/threonine-protein phosphatase 2A 65 kDa regulatory subunit A alpha isoform |
| 111 | P02787 | TF, PRO1400 | Serotransferrin |
| 112 | P05023 | ATP1A1 | Sodium/potassium-transporting ATPase subunit alpha-1 |
| 113 | P50993 | ATP1A2, KIAA0778 | Sodium/potassium-transporting ATPase subunit alpha-2 |
| 114 | P13637 | ATP1A3 | Sodium/potassium-transporting ATPase subunit alpha-3 |
| 115 | P38646 | HSPA9, GRP75, HSPA9B, mt-HSP70 | Stress-70 protein, mitochondrial |
| 116 | P61764 | STXBP1, UNC18A | Syntaxin-binding protein 1 |
| 117 | P02766 | TTR, PALB | Transthyretin |
| 118 | Q71U36 | TUBA1A, TUBA3 | Tubulin alpha-1A chain |
| 119 | P68363 | TUBA1B | Tubulin alpha-1B chain |
| 120 | P68366 | TUBA4A, TUBA1 | Tubulin alpha-4A chain |
| 121 | P07437 | TUBB, TUBB5, OK/SW-cl.56 | Tubulin beta chain |
| 122 | Q9H4B7 | TUBB1 | Tubulin beta-1 chain |
| 123 | Q13885 | TUBB2A, TUBB2 | Tubulin beta-2A chain |
| 124 | Q13509 | TUBB3, TUBB4 | Tubulin beta-3 chain |
| 125 | P04350 | TUBB4A, TUBB4, TUBB5 | Tubulin beta-4A chain |
| 126 | P68371 | TUBB4B, TUBB2C | Tubulin beta-4B chain |
| 127 | P02774 | GC | Vitamin D-binding protein |
| 128 | P07225 | PROS1, PROS | Vitamin K-dependent protein S |
| 129 | P04004 | VTN | Vitronectin |
| 130 | P04275 | VWF, F8VWF | von Willebrand factor |
