# Supplementary material for: Proteomic profile of extracellular vesicles from plasma and CSF of multiple sclerosis patients reveals disease activity-associated EAAT2
Source: J Neuroinflammation. 2024 Sep 2;21:217. doi: 10.1186/s12974-024-03148-x (PMC11370133; doi:10.1186/s12974-024-03148-x)
Supplement: Supplementary file 17 — Additional file 17. [file 12974_2024_3148_MOESM17_ESM.docx]

| plasma EV-EAAT2 | | | | |
| --- | --- | --- | --- | --- |
|  | *RRMS Relapse* | *RRMS Remission* | *SPMS* | *HC* |
| *p-value^a^* | 0,5 | 0,08 | 0,7 | 0,9 |

**Supplementary table 8**. Statistical analysis of EV-EAAT2 plasma level and sex.

Mann Whitney test
